# Fluctuations in EEG band power at subject‐specific timescales over minutes to days explain changes in seizure evolutions

**DOI:** 10.1002/hbm.25796

**Published:** 2022-02-04

**Authors:** Mariella Panagiotopoulou, Christoforos A. Papasavvas, Gabrielle M. Schroeder, Rhys H. Thomas, Peter N. Taylor, Yujiang Wang

**Affiliations:** ^1^ CNNP Lab, Interdisciplinary Computing and Complex BioSystems Group School of Computing, Newcastle University Newcastle upon Tyne; ^2^ Faculty of Medical Sciences Newcastle University Newcastle upon Tyne; ^3^ UCL Queen Square Institute of Neurology, Queen Square London

**Keywords:** band power, chronotherapy, circadian rhythms, cycles, EEG, epilepsy, seizure dynamics

## Abstract

Epilepsy is recognised as a dynamic disease, where both seizure susceptibility and seizure characteristics themselves change over time. Specifically, we recently quantified the variable electrographic spatio‐temporal seizure evolutions that exist within individual patients. This variability appears to follow subject‐specific circadian, or longer, timescale modulations. It is therefore important to know whether continuously recorded interictaliEEG features can capture signatures of these modulations over different timescales. In this study, we analyse continuous intracranial electroencephalographic (iEEG) recordings from video‐telemetry units and find fluctuations in iEEG band power over timescales ranging from minutes up to 12 days. As expected and in agreement with previous studies, we find that all subjects show a circadian fluctuation in their iEEG band power. We additionally detect other fluctuations of similar magnitude on subject‐specific timescales. Importantly, we find that a combination of these fluctuations on different timescales can explain changes in seizure evolutions in most subjects above chance level. These results suggest that subject‐specific fluctuations in iEEG band power over timescales of minutes to days may serve as markers of seizure modulating processes. We hope that future study can link these detected fluctuations to their biological driver(s). There is a critical need to better understand seizure modulating processes, as this will enable the development of novel treatment strategies that could minimise the seizure spread, duration or severity and therefore the clinical impact of seizures.

## Graphical abstract

Epileptic seizures change over time and have been shown to be modulated on circadian or longer timescales. It is an open question whether these changes can be explained by intrinsic fluctuations of each individual patient's physiology. We hypothesize that signal features derived from the continuous iEEG can serve as biomarkers of such intrinsic fluctuations. We find that a combination of iEEG marker fluctuations on different timescales can explain changes in seizure evolutions in most subjects above chance level.

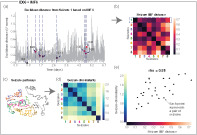

## 1 INTRODUCTION

Epilepsy is a common neurological condition characterised by recurrent, unprovoked seizures (Fisher et al., 2014). It affects approximately 1% of the world's population and a third of patients experience refractory epilepsy, where seizures are not adequately controlled despite medication (Chen, Brodie, Liew, & Kwan, 2018; Kwan & Brodie, 2000).

Importantly, epilepsy is not a static disorder; electrographic seizure and epileptiform activities have been shown to fluctuate over hours to years in both intensity and spatial distribution. Specifically, while seizures often share common features in the same patient (Burns et al., 2014; Kramer et al., 2010; Schevon et al., 2012; Schindler et al., 2011; Truccolo et al., 2011; Wagner et al., 2015), electrographic seizure activity may change in terms of duration (Cook et al., 2016), spatial spread (Karthick, Tanaka, Khoo, & Gotman, 2018; Marciani & Gotman, 1986; Naftulin et al., 2018; Pensel, Schnuerch, Elger, & Surges, 2020), spectral properties (Alarcon, Binnie, Elwes, & Polkey, 1995) from one seizure to the next. Our recent study (Schroeder et al., 2020) has additionally shown that the seizure EEG spatio‐temporal evolution from seizure start to seizure termination (or short: 'seizure evolution') also changes from one seizure to the next in the same patient. Notably, these changes were consistent with daily (circadian) and/or longer‐term fluctuations in most patients (Schroeder et al., 2020). In support of our observations, a recent study quantifying single‐channel properties of seizure onset and offset also noted that different types of dynamics can be seen across different seizures in the same patient (Saggio et al., 2020). Similarly, seizure symptoms are also known to change over time. For example, focal seizures, which evolve into bilateral tonic–clonic seizures, preferentially arise from sleep (Jobst et al., 2001). Subclinical seizures (without clinical symptoms) are also reported to follow circadian patterns (Jin et al., 2017). Finally, seizure severity appears to depend on the severity of the preceding seizure in the same patient (Sunderam, Osorio, & Frei, 2007). Thus, epileptic seizures are not a fully deterministic sequence of abnormal brain activity patterns, but are clearly modulated by processes that shape the neural activity during a seizure and affect seizure severity.

However, it is unclear what these seizure‐modulating processes are, and how to quantify and measure them. Given the evidence of seizure properties fluctuating over various timescales of hours to days, we hypothesise here that the seizure‐modulating processes will also fluctuate over these timescales. From existing literature, we also know that continuously recorded electroencephalograms (EEG) show fluctuations over such timescales. For example, spectral properties of the EEG change from moment to moment (Oken & Chiappa, 1988) and also follow a circadian rhythm (Aeschbach et al., 1999). Global and local characteristics of the continuously recorded (interictal) functional network fluctuate over timescales from hours to days, with circadian rhythm having a particularly strong effect on these dynamics (Geier & Lehnertz, 2017; Geier, Lehnertz, & Bialonski, 2015; Mitsis et al., 2020). Interictalfluctuations related to epilepsy are also seen: high frequency oscillation (HFO) rates vary in location and power within each subject over time (Gliske et al., 2018). Interictal spikes also change in their location and rate over hours to days (Baud et al., 2018; Chen et al., 2021; Conrad et al., 2020; Gliske et al., 2018; Karoly et al., 2016).

We therefore hypothesised that fluctuations of certain features captured in continuously recorded EEG may serve as biomarkers of seizure‐modulating processes. We expected these fluctuations to appear on the timescale of hours to days, and we investigated if they can also explain how seizure evolutions change within the same patient. Previous study suggests that many interictal features, including interictal spike rate (Baud et al., 2018; Karoly et al., 2016, 2017; Proix et al., 2021) and high frequency oscillation rate (Chen et al., 2021; Gliske et al., 2018; Scott, Gliske, Kuhlmann, & Stacey, 2021) may serve as biomarkers for modulatory processes. Here, we investigate the full spectral range, using band power in main EEG frequency bands, to capture a complete view of brain activities. Specifically, we use clustering and dimensionality reduction to detect subject‐specific spectral patterns in continuously recorded EEG. We then extract the temporal fluctuations over minutes, hour, and days in these common spectral patterns and explore whether fluctuations on different timescales are associated with how seizure evolutions change in each subject.

## 2 METHODS

### 2.1 Data acquisition

We analysed open source data from subjects with drug‐resistant focal epilepsy in accordance with the ethical standards set by the Newcastle University Ethics Committee (Ref: 18818/2019). The data consist of a total of 2656 h of long‐term intracranial electroencephalography (iEEG) from 18 subjects (available at http://ieeg-swez.ethz.ch). Continuous recordings in each subject cover 24 to 128 EEG channels and vary between 2 and 12 days. More information about the data is given in Supporting information Section S1. Sampling frequency was either 512 or 1024 Hz depending on the subject. Electrodes (strip, grid and depth) were implanted intracranially by clinicians. The onset and termination of seizures were defined electrographically in the intracranial EEG recordings by visual inspection by an epileptologist for the purpose of subsequent data analysis. The collection of the data was conducted in the Sleep–Wake‐Epilepsy‐Centre (SWEC) at the University Hospital of Bern, Department of Neurology, as part of their presurgical evaluation programme, independently of this study (Burrello, Cavigelli, Schindler, Benini, & Rahimi, 2019).

The iEEG signals were provided in already preprocessed form. Briefly, signals were median‐referenced and band‐pass filtered from 0.5 to 120 Hz using a fourth order Butterworth filter (forward and backward). Seizure onset and termination times were determined by a board‐certified epileptologist. Channels with artefacts were also identified and excluded by the same epileptologist. These steps were all conducted independently of this study and resulted in the publicly available data and annotations. All subjects formally consented to their iEEG data being used for research purposes (Burrello et al., 2019).

### 2.2 IEEG preprocessing

**FIGURE 1:**
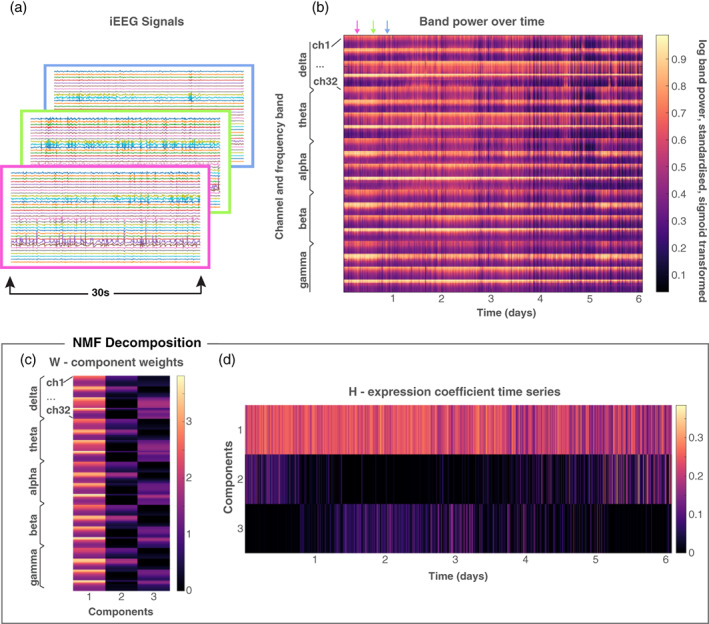
Workflow of data preprocessing; calculation of band power in 30 s epochs and subsequent dimensionality reduction to detect subject‐specific spectral patterns. (a) The multi‐channel continuous iEEG recording was divided into 30 s nonoverlapping epochs. (b) The standardised, log and sigmoid transformed band power. (c,d) NMF (dimensionality reduction) of the band power matrix results in the decomposition *W* × *H*. (c) The matrix *W* contains the basis vectors, each of which had 5× *#channels* weights that represents a pattern of frequency across all channels and frequency bands. (d) The coefficients matrix *H* captures the contribution of each frequency pattern (basis vector) to each time window

We performed additional preprocessing steps to extract iEEG band power from five main frequency bands (Figure 1a). For each recording channel, the signal was divided into 30 s epochs (Figure 1b). For each epoch, the band power was computed for the following frequency bands: *δ*: 1–4 Hz, *θ*: 4–8 Hz, *α*: 8–13 Hz, *β*: 13–30 Hz and *γ*: 30–80 Hz. Band power across the five main frequency bands was estimated using Welch's method for every 30 s epoch, with 3 s sliding window without overlap between consecutive windows. This estimation yielded a time‐varying band power, with each time point corresponding to the mean power within a 30 s window. The band power values were aggregated into band‐specific matrices with dimensions #channels × #epochs. Then, these matrices were log transformed and standardised across all epochs and channels within a frequency band. To enable subsequent analysis steps, we also Sigmoid transformed ($S\left(x\right)={\left[1+\exp\left(-x\right)\right]}^{-1}$) the standardised data to ensure positive entries between 0 and 1. For each subject, we then concatenated the matrices from all frequency bands yielding a single (5 × #channels) × #epochs (henceforth defined as *n* × *T*; Figure 1b). We will refer to this matrix as the data matrix *X* throughout the article.

Note that we did not exclude seizure epochs from the construction of the data matrix, as seizures only represent a few epochs in the context of the continuous recording. Our downstream analysis (with empirical mode decomposition) is robust to 'noise' of this type, and we show in Data S8.2 that this choice does not affect our main results. Note also that our measure of how seizure evolutions change over time (in Section 2.10; Schroeder et al., 2020) was based on functional network activity of the seizure, whilst we used band power features to measure fluctuations of the continuous EEG. Therefore, the fluctuations of the continuous EEG were not trivially related to variability in seizure evolutions (also shown in Supporting information Section S3).

### 2.3 Nonnegative matrix factorization for dimensionality reduction

As the data matrix *X* for each subject is high‐dimensional with redundant information (e.g., in different channels), we applied a dimensionality reduction step on *X* using Nonnegative Matrix Factorization (NMF) (Lee & Seung, 1999). NMF provides a low‐rank approximation to a nonnegative input matrix $X\in {\mathbb{R}}_{+}^{n\times T}$ as the product of two nonnegative matrices, $W\in {\mathbb{R}}_{+}^{n\times k}$ and $H\in {\mathbb{R}}_{+}^{k\times T}$, such that *X* ≈ *W* × *H* ≡ *X* ^*′*^, given an integer *k*. Specifically, we applied the nonnegative singular value decomposition (SVD) with low‐rank correction (NNSVD‐LRC) (Atif, Qazi, & Gillis, 2019), which is a method of low‐rank approximation using an NMF initialisation approach based on SVD.

In this way, we decomposed each subject's band power data matrix *X* into *W* and *H* matrices (Figure 1c,d). Every column of matrix *W* corresponded to a single NMF component and formed a basis vector or feature weight with *n* elements. Each row of *H* represented how a single NMF component evolves over all *T* time epochs. We also refer to a single row of *H* as the NMF‐expression coefficient time series. This dimensionality reduction step not only compressed the data matrix *X* into few relevant dimensions, but can also be understood as a data‐driven pattern detection, or (soft) clustering of recurrent spectral patterns in the continuously recorded EEG. For example, Figure 1 shows that the band power in each channel at a particular time window could be (approximately) described as a weighted sum of three patterns (given by the three basis vectors in *W*). The weights were given as the expression coefficients (in *H*) at each time point. This data‐driven spectral pattern detection essentially provided us with a comprehensive view of the EEG in each subject, without the need to pre‐define specific patterns of interest, which may not acknowledge subject‐specific variations in these spectral patterns.

To determine the optimal number of representative NMF components, *k*, for each subject, we performed NNSVD‐LRC for k=3,4,…,15. For each value of *k*, we obtained the matrices *W* and *H*. Using these matrices, we calculated the relative reconstruction errors $\sum_{n,T}|X-X^{\prime }|/\left(n\times T\right)$, as well as the quantity *c* = *max*{*max*(|*Corr*[*W*]|), *max*(|*Corr*[*H*]|)} for each *k*, where *max*(|*Corr*[*W*]|) represents the maximum absolute correlation among all column pairs of *W*, and *max*(|*Corr*[*H*]| represents the maximum absolute correlation among all row pairs of *H*. The latter represents the strongest correlation or anticorrelation between NMF components in terms of their feature weights *W* and their expression coefficient time series *H*. In this way, redundant information, particularly in *H*, was excluded whilst preserving the important spatio‐temporal patterns for the next processing steps. A distinct number of NMF components, *k*, was selected for each subject. This was the *k* yielding the smallest correlation between the NMF components that had a relative reconstruction error smaller than 5*%*.

After determining the optimal choice of *k*, we obtained two matrices for each subject, *W* and *H*. To re‐iterate, the matrix *W* consists of the basis vectors, while *H* is a multivariate time series with dimensions equal to *k* × *T* (= the number of NMF components × the total number of time epochs).

### 2.4 Extracting fluctuations in interictal band power using MEMD

To investigate fluctuations in band power on different timescales, we analysed the matrix *H* using Empirical Mode Decomposition (EMD) (Huang et al., 1998, 2003). It is well known that EEG signals are nonstationary processes characterised by time‐varying features (Fingelkurts & Fingelkurts, 2001; Kaplan, Fingelkurts, Fingelkurts, Borisov, & Darkhovsky, 2005). EMD is a popular data‐adaptive method to detect nonstationary and nonrhythmic fluctuations on different timescales. Compared to Fourier and Wavelet‐based approaches, EMD does not assume any particular basis function or local stationarity. EMD also does not require detrended time series and does not make assumptions about trends or timescales of trends. It has the advantage of fully decomposing the signal into the full range of timescales of fluctuations; their point‐wise summation fully reconstructs the original signal. As the nature of these band power fluctuations is unknown and most likely not stationary, we opted for a data‐driven method that makes as few assumptions as possible.

EMD decomposes an input signal *Y* (*t*), into *M* finite narrow‐band fluctuations, known as intrinsic mode functions (IMFs), based on the local extrema of the signal: $Y\left(t\right)=\sum_{i=1}^{M}{\mathrm{IMF}}_{i}\left(t\right)+r\left(t\right)$, where *r*(*t*) is the residue signal (Huang et al., 1998). The IMFs additionally satisfy the properties that make the Hilbert‐transform well‐defined and therefore naturally yield instantaneous frequency and phases for each IMF.

However, local extrema are not directly applicable to multivariate time series signals (Rehman & Mandic, 2010), as we have in the *H* matrix. Therefore, we used an extension of the EMD to multi‐dimensional space, called the Multivariate Empirical Mode Decomposition (MEMD) (Rehman & Mandic, 2010). In MEMD, multiple projections of the multivariate signal are generated along different directions in *n*‐dimensional spaces; the multidimensional envelope of the signal is then obtained by interpolating across the different envelopes of these projections (Rehman & Mandic, 2010). An additional advantage of this method is that it yields the same number of IMFs across the different dimensions of the multivariate signal, and preserves fluctuations of similar frequency across the different dimensions within each of the IMFs (mode‐alignment; Rehman & Mandic, 2010).

For the purpose of this analysis, we used MEMD to decompose the NMF‐expression coefficient time series, *H*, into a number of multi‐dimensional oscillatory modes. Therefore, the matrix *H* can be represented by the sum of *M* multi‐dimensional IMF signals, where the dimension for each IMF is equal to *k* (i.e., the number of rows of the matrix *H*, which corresponds to the number of NMF components). To clarify, we can think of all IMFs in a specific dimension as the decomposition of the corresponding NMF‐expression coefficient time series. Thus, IMF_*i*,*j*_ refers to the *j*‐th dimension of the *i*‐th IMF timescale. The *j*‐th NMF‐expression coefficient time series *H*_*j*_ = *Y*_*j*_(*t*) can be written as $Y_{j}\left(t\right)=\sum_{i=1}^{M}{\mathrm{IMF}}_{i,j}\left(t\right)+r_{j}\left(t\right)$. This equation applies to every NMF component j=1,…,k.

### 2.5 Extracting time‐varying characteristics from band power fluctuations (IMFs) using Hilbert spectral analysis

To obtain a time‐frequency representation of the oscillatory modes (IMFs), and hence derive their time‐varying characteristics (instantaneous frequency, phase, and amplitude), we applied a Hilbert‐transform on each dimension of the IMF (following classical analysis methods for EMD) (Huang, 2014; Huang et al., 1998, 2003).

For any (real‐valued) univariate signal *u*(*t*), we can define its Hilbert transform as:

(1) $$H\left(u\right)\left(t\right)=\frac{1}{\pi }P\int_{-\infty }^{+\infty }\frac{u\left(\tau \right)}{t-\tau }\mathrm{d\tau},$$

where *P* represents the Cauchy principal value for any function *u*(*t*) ∈*L* ^*P*^ class (Huang et al., 1998).

The analytical signal *v*(*t*) obtained from the Hilbert transform can be expressed as:

(2) $$v\left(t\right)=u\left(t\right)+\mathrm{iH}\left(u\right)\left(t\right)=a\left(t\right)e^{\mathrm{i\theta}\left(t\right)},$$

where

(3) $$a\left(t\right)=\sqrt{u{\left(t\right)}^{2}+H\left(u\right){\left(t\right)}^{2}}$$

and

(4) $$\theta \left(t\right)=\tan^{-1}\left(\frac{H\left(u\right)\left(t\right)}{u\left(t\right)}\right)$$

are the instantaneous amplitude and instantaneous phase, respectively.

The instantaneous frequency, *f*(*t*), can then be calculated as follows:

(5) $$f\left(t\right)=\frac{\mathrm{d\theta}\left(t\right)}{\mathrm{dt}}.$$

The application of EMD along with Hilbert transform leads to the so‐called Hilbert‐Huang transform. Through the Hilbert spectral analysis, each IMF's instantaneous frequency can be represented as functions of time. The result is a frequency‐time distribution of signal amplitude (or energy using the squared values of amplitude, *a* ^2^(*t*)), designated as Hilbert amplitude spectrum or Hilbert spectrum (or Hilbert energy spectrum if energy is used instead of amplitude), *H*(*f*, *t*).

For each univariate IMF signal, we can obtain the Hilbert energy spectrum as a function of instantaneous frequency and time mathematically using the following formula:

(6) $$H\left(f,t\right)=\left\{\begin{array}{ll} a^{2}\left(t\right), & f=f\left(t\right)\\ 0, & \text{otherwise}. \end{array}\right.$$

For visualisation purposes, we will display the inverse of the instantaneous frequency, that is, the instantaneous period length, also termed 'cycle length' in the following.

The Hilbert‐Huang marginal spectrum *h*(*f*) of the original signal *u*(*t*) can then be defined as the total energy distributed across the frequency space within a time period [0, *T*]. Mathematically, this definition can be expressed as shown below:

(7) $$h\left(f\right)=\int_{0}^{T}H\left(f,t\right)\mathrm{dt}.$$

By using Equations (6) and (7), we can obtain the Hilbert‐Huang marginal spectrum for a univariate IMF signal. However, the application of the multivariate EMD results in multivariate IMF signals. In order to compute the Hilbert‐Huang marginal spectrum of each multivariate IMF signal across all dimensions, we simply averaged over the dimensions *H*_*i*_(*f*, *t*) across i=1,…,k dimensions:

(8) $$\bar{h}\left(f,t\right)=\frac{\sum_{i=1}^{k}H_{i}\left(f,t\right)}{k}.$$

The corresponding marginal spectrum $\bar{h}(f)$ was then similarly defined as:

(9) $$\bar{h}\left(f\right)=\int_{0}^{T}\bar{H}\left(f,t\right)\mathrm{dt}.$$

**FIGURE 2:**
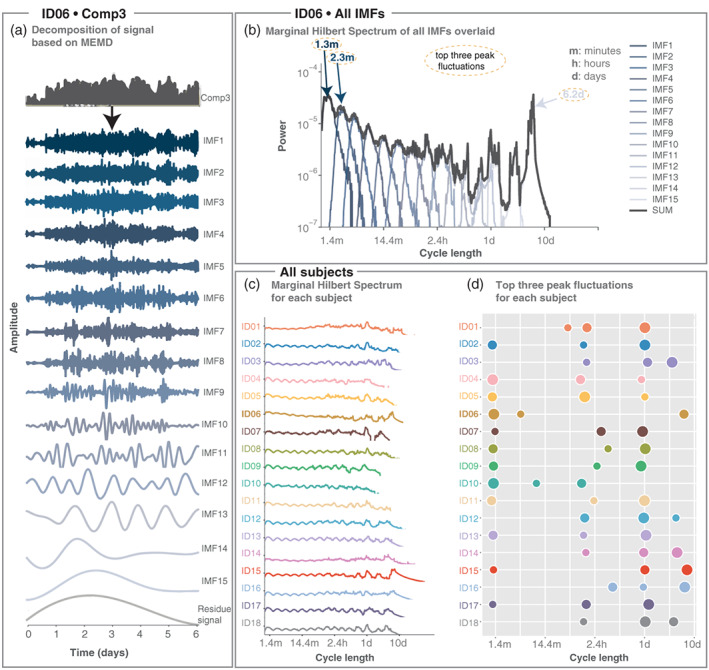
MEMD detects fluctuations on different timescales for each subject. (a) MEMD yields 16 IMFs in example subject ID06. Only one dimension of the IMF (corresponding to the first NMF component) is shown for simplicity. IMFs are presented in ascending order (fastest to slowest, top to bottom). The last trace is the residue signal. (b) Marginal Hilbert spectrum for all IMFs aggregated across all dimensions in example subject ID06. The black line represents the Marginal Hilbert frequency spectrum across all IMFs. The x‐axis shows the instantaneous period length (inverse of instantaneous frequency), which we also termed 'cycle length'. Top three peak fluctuations are indicated with arrows. (c) Marginal Hilbert frequency spectrum across all IMFs for each subject. (d) Bubble plot of peak fluctuations for the three highest power densities according to the Marginal Hilbert frequency spectrum across all IMFs for each subject. The size of the bubbles indicates the first, second, and third peak in descending order

For numerical computations, we discretised time *t* to compute the integrals as sums. Figure 2 shows the marginal Hilbert‐Huang spectra for different multivariate IMFs in an example subject.

### 2.6 Peak fluctuation frequency in each IMF

Within each subject, each IMF was characterised by a peak fluctuation frequency (measured in cycles/day here). It was determined as the frequency with the highest power based on the marginal Hilbert‐Huang spectrum over all frequencies, $\bar{h}(f)$.

#### 2.6.1 Finding a circadian IMF

We will later focus one part of our analysis on IMFs that fluctuate on the timescale of 24 h (1 cycle/day). To detect those IMFs, we found IMF(s) with a peak fluctuation frequency of 1 cycle/day. If two IMFs were found (i.e., both displayed the a peak frequency at around 1 cycle/day), then the circadian IMF was determined to be the IMF with the higher power. This case only occurred in one subject.

### 2.7 Relative contribution of iEEG main frequency bands in different IMFs

To understand how much each of the iEEG frequency bands and channels contributed to a certain IMF, we first determined how much each dimension of the IMF contributed to the overall power of the IMF. To this end, we first obtained the mean power *E*_*ij*_ in each dimension *j* of every *i*‐th IMF signal:

(10) $$E_{\mathrm{ij}}=\frac{\sum_{t=0}^{T}a_{\mathrm{ij}}{\left(t\right)}^{2}}{T},$$

where *T* is, as before, the number of time epochs and *a*_*ij*_(*t*) is the instantaneous amplitude for the *j*‐th dimension of *i*‐th IMF signal at time point *t*. One of the main properties of MEMD is that multivariate signals are decomposed into multivariate IMF signals of the same dimensions, where all dimensions within an IMF share fluctuations of the same timescale (Lv, Yuan, & Song, 2016). Hence, focusing on the mean power over time of each dimension within an IMF is a good indication of the power on a particular timescale. The relative contribution of each *j*‐th dimension to the *i*‐th IMF (or relative power) was then defined as:

(11) $$R_{\mathrm{ij}}=\frac{E_{\mathrm{ij}}}{\sum_{j=1}^{k}E_{\mathrm{ij}}},$$

with *k* indicating the number of IMF dimensions (also the number of NMF components).

**FIGURE 3:**
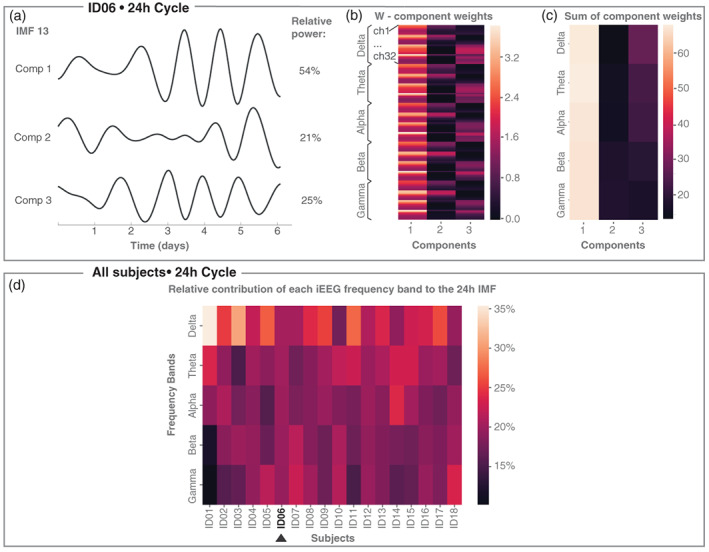
Contribution of iEEG main frequency bands to the circadian IMF. (a) IMF 13 in example subject ID06 shows circadian fluctuations across all three dimensions, each of which corresponds to an NMF component. Dimension 1 shows the highest relative power in this IMF. (b) W component weight matrix (same as Figure 1c). (c) The sum of the component weights across all channels within each frequency band. (d) Contribution of each iEEG frequency band to the circadian IMF across all subjects obtained by forming the sum over the matrix in (c) weighted by the relative power in (a). To be able to compare subjects to each other, each column here has been normalised to form a percentage contribution

Using the relative contribution of each dimension as weights, we can then form the weighted sum of all dimensions in terms of contributions of iEEG main frequency bands. By summing channel contributions for each iEEG main frequency band (see Figure 3b&c for an example subject), we obtained a matrix of dimensions (# main frequency bands = 5) × (# dimensions = *k*). This matrix was then multiplied with the weight indicating the contribution of each dimension to yield a vector (of length # main frequency bands = 5) representing the contribution of each main frequency band to particular IMF for each subject (Figure 3d).

### 2.8 Different band power fluctuations reveal spatial heterogeneity within iEEG main frequency band

To determine if all recording channels contribute homogeneously to an IMF in a particular frequency band, we used a measure that quantifies sparsity of a distribution: the *Gini index* (Hurley & Rickard, 2009). Given a vector $x=\left(x_{1},x_{2},\ldots ,x_{N}\right)$ sorted in ascending order such that $x_{1}<x_{2}<\ldots <x_{N}$, the Gini index can be derived using the following formula:

(12) $$G\left(x\right)=1-2\sum_{i=1}^{N}\frac{x_{i}}{{\left\|x\right\|}_{1}}\left(\frac{N-i+\frac{1}{2}}{N}\right).$$

It can range from 0 to 1, with values closer to 0 indicating low sparsity (homogeneity) and values closer to 1 corresponding to higher sparsity (heterogeneity).

We derived the Gini index for each IMF across different channels within each main frequency band. In other words, for each IMF, we first computed the contribution *C*_*i*_ to each *i*‐th IMF as the product of the relative power (Equation 11) and the weights matrix: $C_{i}=\sum_{j}R_{\mathrm{ij}}\times W_{j}$, with *i* indexing the IMF number, and *j* indexing its dimension. Specifically, *W*_*j*_ is the *j*‐th column of the *W* matrix from the NMF decomposition (Figure 1c), whereas *R*_*ij*_ is a scalar representing the relative power of the *j*‐th dimension to the *i*‐th IMF (see Section 2.7). The resulting *C*_*i*_ is a vector of length #frequency bands (= 5) × #channels, that is, the same length as *W*_*j*_. As we are interested in the distribution of each *C*_*i*_ across channels for each frequency band, we applied the Gini index to each frequency band separately in each *C*_*i*_, yielding one Gini index per frequency band and IMF.

### 2.9 Seizure distance in terms of a particular band power fluctuation (IMF)

For each subject, we quantified the difference between pairs of seizures in terms of each IMF. This measure (which we subsequently term the 'IMF distance') thus quantifies how different two seizures are to each other in terms of a particular fluctuation of the band power. To obtain this difference, we first computed the product *W* × IMF_*i*_(*t*), where IMF_*i*_(*t*) is the multi‐dimensional *i*‐th IMF (*k* × *T* matrix). The product yields the matrices $X_{{\mathrm{IMF}}_{i}}^{\prime }$ for all i=1,…,M timescales. $X_{{\mathrm{IMF}}_{i}}^{\prime }$ reconstructs the *i*‐th IMF in the original space of all channels and frequency bands. For each $X_{{\mathrm{IMF}}_{i}}^{\prime }$, we computed a distance matrix based on the multivariate Euclidean distance of IMF values for each pair of seizures: $D_{i}\left(a,b\right)=\|X_{{\mathrm{IMF}}_{i}}^{\prime }\left(t_{a}\right)-X_{{\mathrm{IMF}}_{i}}^{\prime }\left(t_{b}\right)\|$, where *t*_*a*_ and *t*_*b*_ are the time epochs of the seizure pair's onset. Therefore, we obtained *M* IMF seizure distance matrices per subject, each representing the pairwise seizure distance for a specific IMF.

Note that any seizure‐induced changes in the band power will only be present in a few epochs (as we use 30 s long epochs). Therefore, the seizures are considered to only influence the fastest IMFs (highest‐frequency fluctuations), while they have little effect on the slower IMFs. Supporting information Section S8.1 additionally shows that our main results were reproduced by using the IMF seizure distances obtained from one epoch before the seizure onset epoch (*t*_*a*_ − 1 and *t*_*b*_ − 1).

### 2.10 Quantifying differences in seizure evolutions using seizure dissimilarity

To quantify how seizures themselves change over time in terms of the seizure EEG evolutions, in our previous study, we introduced a quantitative measure of how dissimilar two seizures are within a subject (Schroeder et al., 2020). Briefly, each epileptic seizure in a subject was analysed in terms of its evolution through the space of functional network dynamics (using exactly the same pipeline as (Schroeder et al., 2020)). Each pair of seizures was then compared to each other using dynamic time warping (Sakoe & Chiba, 1978), allowing us to recognise seizures with shared evolutions (or parts of evolutions), even if the seizures evolved and different rates. The average distance between the warped seizures was then taken as the dissimilarity measure. As such, for each subject, we obtained a 'seizure dissimilarity' matrix, which captures the pairwise dissimilarity between the subject's seizure evolutions.

### 2.11 Association between seizure dissimilarity & IMF seizure distance

Finally, we related how seizure evolutions changed over time (quantified using seizure dissimilarity) with fluctuations seen in the continuously recorded iEEG (quantified using IMF seizure distances). In subjects with at least six recorded seizures, we investigated if IMF seizure distances were associated with seizure dissimilarity. For every subject, we used a linear regression framework, where the seizure dissimilarity was the response variable and the IMF seizure distances were the explanatory variables. The observations were the entries of the seizure dissimilarity matrix and IMF seizure distance matrix. As each matrix was symmetric, we only used the upper/lower triangular elements. We also included the EMD residue signal distances, and temporal distances of seizures (how far apart in time each pair of seizures occurred) as additional explanatory variables. The response, as well as the explanatory variables, were standardised individually before fitting the model.

We performed a variable selection step for our analysis, as the number of explanatory variables (i.e., *M* + 2) was relatively large. We used LASSO (Least Absolute Shrinkage and Selection Operator; Tibshirani, 1996), which is a sparse shrinkage method. Linear regression coefficients were calculated based on least squares, subject to the *L*_1_ penalty. The LASSO also accounted for any collinearity issues between variables. As we were interested in detecting positive relationships between the response variable (as these were distances) and the explanatory variables, we used a constrained positive LASSO; that is, coefficients were constrained to be nonnegative. For the LASSO, the tuning parameter *λ* was selected using a 10‐fold cross validation method from a range of values $\lambda =10^{-3},10^{-2.95}\ldots ,10^{1.95},10^{2}$ (see Supporting information Figure S6.0.1).

After selecting a small number of explanatory variables, an ordinary least squares regression was performed for each subject to obtain *R* ^2^ and 95*%* confidence intervals for the coefficients.

### 2.12 Statistical analysis

To assess if the level of explanatory power of the best model selected for each subject has occurred by chance, we performed two separate tests of statistical significance for the adjusted *R* ^2^. Both test yielded very similar results and are shown in Supporting information Section S4.

In the first test, we randomly selected seizure onset times by generating a sample from the uniform distribution on the interval (0, *T*) over 500 iterations. The size of the sample was equal to the number of annotated seizures for each iteration. Then, keeping the randomly picked seizure onset times unsorted, we obtained for each one of them new IMF seizure distance matrices and performed the LASSO and linear regression, as described in the previous section, leaving the response variable unchanged. Finally, we calculated the adjusted *R* ^2^ for each iteration. Across all iterations, the adjusted *R* ^2^ values were used to estimate the distribution of the test statistic used in the permutation test. P‐values were then calculated as the percentage of adjusted *R* ^2^ values that were larger in the permutation distribution. Statistical significance was determined based on a significance level of 5*%*.

In the second test, we permuted the order of the seizures without permuting the seizure timing over 500 iterations. We then performed the LASSO and subsequent steps as in the first test.

### 2.13 Data and code availability

The long‐term iEEG recordings for all subjects are available at http://ieeg-swez.ethz.ch/ under the section 'Long‐term Dataset and Algorithms' (Burrello et al., 2019).

Initial signal processing was performed using Matlab version 2019a and Matlab's built‐in functions. NMF and MEMD were implemented using the following publicly available functions:

- Nonnegative matrix factorisation was conducted using the NNSVD‐LRC function from https://sites.google.com/site/nicolasgillis/code (Atif et al., 2019).
- Multivariate empirical mode decomposition was applied using code from http://www.commsp.ee.ic.ac.uk/~mandic/research/emd.htm (Rehman & Mandic, 2010).

For the remainder of the analysis and the construction of all figures, we used Python version 3.5. Either standard functions obtained from published libraries supported by Python were used or custom code written in Python. The main functions used in the analysis are listed below:

- Hilbert transform: scipy.signal.hilbert
- LASSO: sklearn.linear_model.Lasso
- k‐fold cross‐validation: sklearn.model_selection.kFold
- Multiple Linear Regression: statsmodels.api.ols

Our analysis code and data (post processing) can be found on https://dx.doi.org/10.5281/zenodo.5798022.

## 3 RESULTS

We analysed fluctuations in band power for 18 subjects with focal epilepsy. We investigated if fluctuations on specific timescales were driven by particular iEEG frequency bands or spatially localised activity. We then explored if these temporal fluctuations were associated with how seizures change within subjects.

### 3.1 iEEG band power patterns fluctuate on different timescales

After extracting band power in the main frequency bands (*δ*, *θ*, *α*, *β*, *γ*) in 30 s nonoverlapping sliding windows for each iEEG channel (Figure 1a,b), we performed dimensionality reduction using nonnegative matrix factorisation (NMF) approach. NMF effectively grouped channels and frequency bands to form components that represent specific band power patterns. Weights for channels and frequency bands in each component are shown as columns in matrix *W*, Figure 1c. The expression coefficients of these components at each time point was then given by the *H* matrix, which essentially yielded a time series for each component (Figure 1d). The weight represented a subject‐specific pattern of EEG band power activity across channels, and the strength of expression of this pattern at any given time point was given by the expression coefficients. In short, the set of coefficient time series (rows in *H*) indicated the fluctuations of subject‐specific EEG spectral patterns over time.

For each subject, we then used Multivariate Empirical Mode Decomposition (MEMD) to determine the different fluctuations on different timescales for each NMF coefficient time series. Figure 2a shows the MEMD results for a single NMF component in example subject ID06, yielding 15 Intrinsic Mode Functions (IMFs) and a residue signal. Faster IMFs (e.g., IMF1, 2 and 3) are often thought to contain noise, but might also reflect genuine fluctuations in the initial signal, such as cyclic alternating pattern (Parrino, Grassi, & Milioli, 2014). For simplicity, we retained all IMFs for the subsequent main results and refer the reader to Supporting information Section S7 for a more detailed analysis of noisy IMFs based on permutation test.

Using the instantaneous frequency and amplitude through the Hilbert transform, we obtained the marginal spectral densities of each IMF in each dimension. Figure 2b shows the marginal spectral densities averaged across all dimensions for each IMF (blue lines) for example subject ID06. Some distinct peaks are seen especially in the slower IMFs, for example, IMF13 (at cycle length of ≈ 1 day), IMF14 (at cycle length of ≈ 3.3 days), IMF9 (cycle length ≈ 3 h), IMF8 (cycle length ≈ 1.6 h), etc. Note that both EMD and MEMD essentially act as dyadic filter banks (Flandrin, Rilling, & Goncalves, 2004; Ur Rehman & Mandic, 2011; Wu & Huang, 2004); thus, the dyadic pattern seen in the faster IMFs is not surprising. Supporting information Section S7 shows the marginal spectral densities corrected for potential noise fluctuations.

As expected from previous literature (Baud et al., 2018; Karoly et al., 2016), we found that all subjects displayed circadian band power fluctuations (Figure 2c). The presence of these circadian fluctuations helps validate our approach for extracting relevant timescales in interictal fluctuations. Meanwhile, fluctuations on other timescales were more subject‐specific in cycle length. For 10 out of 18 subjects (ID01, ID02, ID07, ID08, ID09, ID11, ID12, ID13, ID17 and ID18) the circadian fluctuation had the highest density (Figure 2d). For six subjects (ID03, ID04, ID05, ID14, ID15 and ID16) the circadian fluctuation was slightly lower in density, as the highest density was seen in slower or faster IMFs. For two subjects (ID06 and ID10) the circadian fluctuation did not feature in the top three highest densities, but a peak at 1 cycle per day can still be observed in ID06 (Figure 2c).

### 3.2 All iEEG frequency bands contribute to the circadian IMF

Following the observation of a circadian fluctuation in all subjects, we assessed the contribution of each iEEG frequency band to the circadian IMF. We first determined the circadian IMF, which was IMF 13 in example subject ID06 (Figure 3a). We then calculated the relative power in each dimension of the IMF, each of which corresponded to an NMF component. For example, in subject ID06, the majority of its power (54%) was concentrated in dimension 1 (Figure 3a). We also noted that the circadian fluctuation did not follow the same phase in all dimensions of the IMF, potentially indicating the presence of multiple processes fluctuating on a circadian timescale. Since we are interested in the overall contribution of each frequency band to the circadian cycle, we decided to assess the contribution of different frequency bands over all dimensions next.

From the dimensionality reduction step, we had already obtained the weights across all iEEG frequency bands and channels (matrix W, see Figure 3b). For each NMF component, we computed the weight of each frequency band by summing the weights of that frequency band across all channels (Figure 3c). Finally, a sum weighted by the relative power in the IMF over all dimensions was obtained representing the relative contribution of each frequency band to the IMF. For most subjects, *δ* band power contribution was slightly higher compared to the other frequency bands for the circadian IMF. However, other frequency bands also contributed to the circadian IMF in most subjects (Figure 3d).

### 3.3 Subsets of channels contribute to multidien band power fluctuations

**FIGURE 4:**
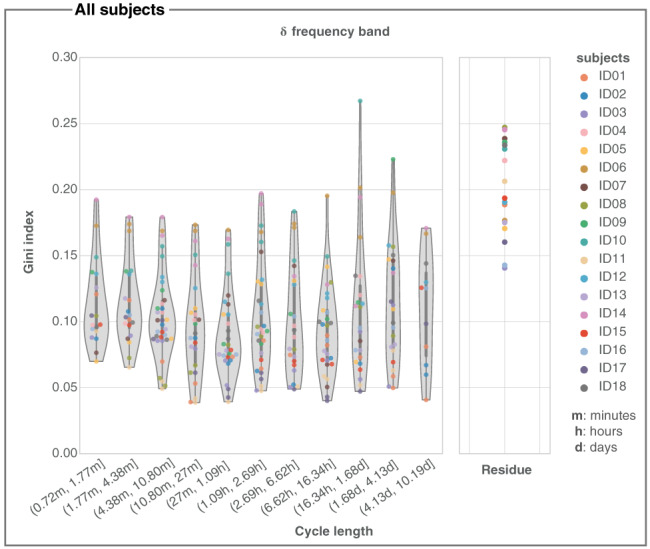
Gini index of IMFs for the *δ* frequency band across all subjects. Across all subjects, we grouped IMFs based on their peak IMF cycle length and show the distribution of the corresponding Gini indices as a violin plot with enclosed box plot. The thick grey bar represents the inter‐quartile range. For visualisation, we converted the peak frequency to cycle length (*x*‐axis). The residue is shown separately

Within each frequency band we also investigated the contribution of each channel to an IMF. Specifically, we investigated if the contributions were heterogeneous across channels. We used the Gini index as a measure of spatial heterogeneity, where 0 (1) indicates a completely homogeneous (heterogeneous) channel contribution for each IMF. Figure 4 shows the distribution of Gini indices of all IMFs in the *δ* band across all subjects, where IMFs are grouped by the IMF peak frequency. Results for other iEEG main frequency bands are similar and shown in Figure S5.0.1. Overall, the Gini indices are low for all IMFs, indicating that IMFs are not driven by a small group of channels. However, there is a clear tendency for long‐term (multidien) trends to display a higher Gini index, indicating that a subsets of channels may contribute more to those.

### 3.4 Band power IMF fluctuations are associated with seizure dissimilarity in most subjects

As the final part of our analysis, we investigated if these fluctuations on different timescales influenced, or modulated, changes in seizure evolutions over time in individual subjects. Particularly, we previously showed that seizure network evolutions change over time in every subject, and that these changes could be explained by hypothetical circadian or longer timescale modulators (Schroeder et al., 2020). Hence, we explored if the subject‐specific fluctuations represented by the IMFs were associated with changes in seizure evolutions.

**FIGURE 5:**
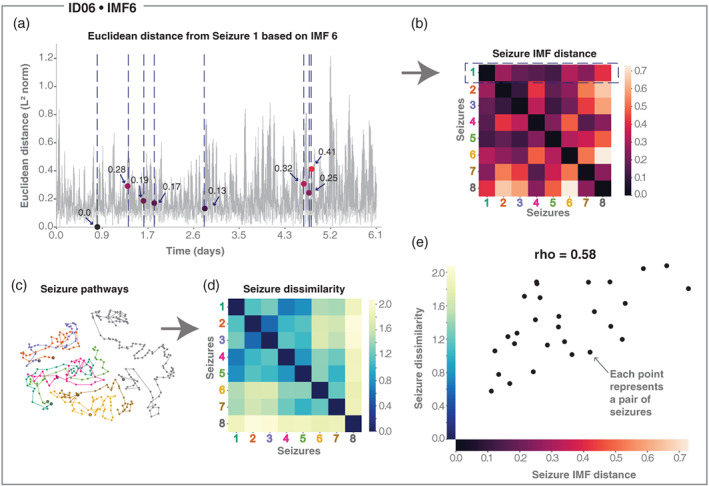
Relating seizure dissimilarity and IMF seizure distance. Throughout the figure we use example subject ID06 and IMF6. (a) Euclidean distance of all time points to the first seizure in terms of IMF6. Blue dashed vertical lines indicate seizure timing. Dots mark the value of the IMF distance to the first seizure and colours of dots correspond to the colour scale in (b). (b) Seizure IMF distance matrix for IMF6. The first row is a representation of the data in (a). (c) Visualising seizure evolutions as pathways (see Supporting information Section S2 and Schroeder et al., 2020). Seizures are displayed with distinct colours to distinguish seizure events. The starting points of seizures are marked with a black outline circle. In this projection, parts of seizures with similar network evolutions tend to be placed closer together, and seizures with similar evolutions will therefore approximately overlap (e.g., orange and purple pathways). (d) Seizure dissimilarity matrix, capturing the differences in seizure evolutions over time between each pair of seizures. (e) Scatter plot of seizure dissimilarity and the seizure IMF distance (Spearman's correlation, *ρ* = 0.58)

For each IMF in each subject, we first determined their corresponding seizure IMF Euclidean distance matrix (Figure 5a,b). For example, in subject ID06's IMF6, we calculated the Euclidean distance of every time point to the time point of the first seizure (Figure 5a) across all dimensions. By reading out all the Euclidean distances to all the other seizure time points, we obtained the first row of the seizure IMF Euclidean distance matrix (Figure 5b). The same process was repeated for all other seizures in this subject. This distance matrix had dimensions of number of seizures by number of seizures and represented how different the IMF state was for each seizure pair.

By using the same techniques as in (Schroeder et al., 2020), we obtained a seizure dissimilarity matrix, which expressed the dissimilarity of each pair of seizure evolutions through the space of network dynamics (Figure 5c,d). The seizure dissimilarity matrix thus quantified how much each pair of seizures differed within a subject. By relating the set of seizure dissimilarities to the corresponding set of IMF Euclidean distance, we investigated if there was an association between changes in seizure evolutions and interictal band power fluctuations (Figure 5e).

**FIGURE 6:**
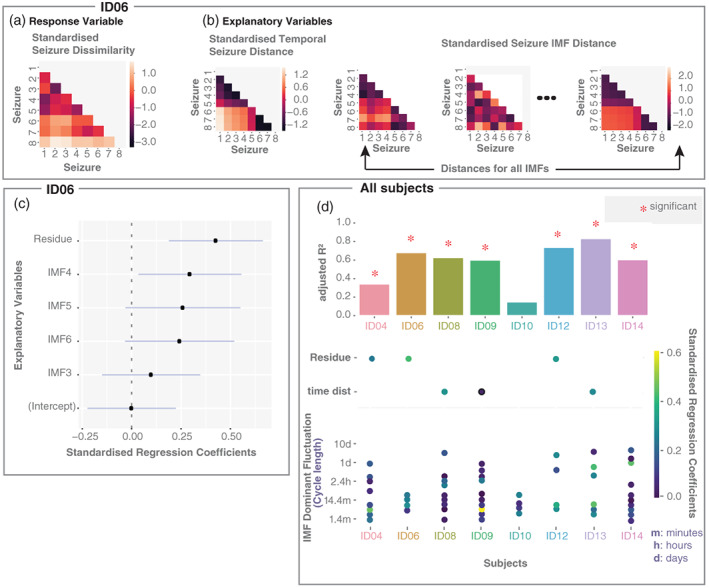
A combination of IMF seizure distances on different timescales can explain seizure dissimilarity in most subjects in a multiple regression model. (a) Standardised seizure dissimilarity matrix (response variable). Only the lower triangle of the symmetric matrix is shown, where each entry serves as an observation. (b) Explanatory variables: The matrix on the left shows the standardised temporal seizure distance. Each entry corresponds to the absolute time difference between seizures. The remaining matrices are standardised seizure IMF distance matrices. (c) Coefficient estimates (black dots), based on ordinary least squares regression for subject ID06, with lines indicating 95% confidence intervals. Only five explanatory variables were left after performing variable selection based on constrained LASSO. (d) Summary across subjects basedon Ordinary Least Squares (OLS) models with explanatory variables obtained by the constrained LASSO. Top: Bar chart of the adjusted *R* ^2^. Red stars indicate *p* values ≤0.05. Bottom: Scatter plot indicating the OLS coefficient estimates for the residue, temporal distance (when these variables remained in the model), together with explanatory IMFs and their corresponding IMF peak frequency for each subject. For visualisation, we converted the peak frequency to cycle length

To generalise this approach to all IMFs in a subject, we fitted a multiple linear regression model, where the sets of seizure IMF distances (derived from different IMFs) were explanatory variables and the seizure dissimilarity was the response variable (Figure 6a). We also included the EMD residue signal and temporal distance between seizures (i.e., how far apart in time each seizure pair occurred) as explanatory variables to model fluctuations of longer timescales than the recording time. The observations were pairs of seizures. After LASSO variable selection and linear regression, the estimated regression coefficients for example subject ID06 are shown in Figure 6c. For this particular subject, the strongest explanatory effect (as measured by the standardised regression coefficients, also know as beta‐weights) was seen in the EMD residue signal followed by some faster IMFs (IMF [Cycle length]: IMF3 [4 min], IMF4 [7.5 min], IMF5 [15 min] and IMF6 [26 min]). According to the model, 67.42*%* of the variability in seizure dissimilarities was explained by explanatory variables (i.e., adjusted *R* ^2^ = 0.6742).

Across subjects, we fitted the multiple linear regression model only for subjects with at least six seizures, resulting in eight subjects with analysed seizure evolutions. Out of the eight subjects, six had an adjusted *R* ^2^ around or above 0.6 (Figure 6d). Figure S4.1.1 additionally shows that the adjusted *R* ^2^ values would have not occurred by chance in any subject except for ID10. For six out of eight subjects, circadian IMFs were also part of the explanatory variables (Figure 6d). Ultradian IMFs also tended to remain as explanatory variables in the models for all subjects. Temporal distance between seizures remained as an explanatory variable in three subjects, and the residue signal also remained as an explanatory variable in three additional subjects. Overall, a subject‐specific combination of different fluctuations was provided a good explanation of seizure variability in most subjects.

Note that band power fluctuations are not expected to trivially correlate with how seizures change, as (i) the seizure network evolutions changes are detected on a finer timescale (seconds) using a functional network measure of the time series rather than a spectral property; (ii) seizure onset network patterns (as measured by functional networks) are also expected to differ substantially from pre‐ictal network patterns (Shah et al., 2019); (iii) the impact of seizures on the band power fluctuation are most likely to be limited to one or few 30 s windows and hence also likely to be limited to the fastest IMF only. In Supporting information Section S3, we show that the band power without being decomposed into different timescales does not explain how seizures change, indicating that our results did not arise from trivial associations between seizure evolutions and their corresponding interictal periods. In Supporting information Section S8.1, we also reproduced our results using the pre‐ictal (one 30 s window ahead of the seizure) band power fluctuations, which were not impacted by seizure evolutions.

## 4 DISCUSSION

We analysed fluctuations in subject‐specific iEEG band power patterns over time and found that these patterns fluctuate over a wide range of timescales (from minutes to days), including a strong circadian fluctuation in most patients. A subject‐specific combination of these fluctuations provided a good explanation (adjusted *R* ^2^ ≥ 0.6) for how seizure EEG spatio‐temporal evolutions change from one seizure to the next within the same subject. Based on these findings, we suggest that band power fluctuations in continuously recorded EEG may be a marker of modulators of seizure activity.

Fluctuations on various timescales of the continuous EEG have been reported in several studies using iEEG recordings. The prevalence of a strong circadian rhythm in EEG patterns has long been known (Aeschbach et al., 1999; Cummings, Dane, Rhodes, Lynch, & Hughes, 2000; Scheich, 1969; Smyk & van Luijtelaar, 2020; Spencer et al., 2016). Weaker ultradian (more than 1 cycle per day) rhythms have been reported in long‐term EEG band power (Chapotot, Jouny, Muzet, Buguet, & Brandenberger, 2000; Kaiser, 2008) and functional connectivity (Mitsis et al., 2020). Subject‐specific multidien (multi‐day, i.e., <1 cycle per day) rhythms have also been detected in for example, the rate of interictal epileptiform activity (Baud et al., 2018; Karoly et al., 2016), and the variance and autocorrelation of EEG signals (Maturana et al., 2020). In agreement, we observed the circadian cycle in all subjects and additional fluctuations on ultradian and multidien timescales that were subject‐specific.

These fluctuations of EEG features on different timescales most likely reflect biological processes. However, the mapping from EEG biomarkers to underlying time‐varying processes is incomplete. Various hypotheses exist regarding the interpretation of these EEG fluctuations (Bernard, 2021; Karoly et al., 2021; Rao, Leguia, Tcheng, & Baud, 2021), and their possible drivers (Badawy, Freestone, Lai, & Cook, 2012; Karoly, Rao, et al., 2021; Meisel et al., 2015; Payne et al., 2021; Rakers et al., 2017) include hormonal and metabolic cycles, changes in antiepileptic medications, and external influences such as the weather. In this study, we therefore took a subject‐specific data‐driven approach that allowed us to detect any prominent fluctuations, regardless of their subject‐specific source. Future study will explore a wider range of EEG biomarkers and elucidate the exact mapping between different fluctuations and the underlying physiological or pathological processes.

Additionally, we make two observations about band power fluctuations on different timescales. First, we saw that different frequency bands appeared to contribute a similar amount to the circadian fluctuation of iEEG band power, although subtle subject‐specific patterns of contribution are also noted. However, our analysis was performed across all dimensions of our data. The different dimensions of the IMF can display phase and amplitude differences (e.g., Figure 3a), indicating that different circadian fluctuations (with different phases) exist in each subject, as has been reported before (Aeschbach et al., 1999). Future study may wish to investigate the frequency contributions to different dimensions of IMFs and also relate those IMFs to other physiological variables such as body temperature or plasma melatonin (Aeschbach et al., 1999).

The second observation is that slower (multi‐day fluctuations, and slow trends) tended result from changes in subsets of channels, whereas faster (circadian and ultradian) fluctuations tended to arise as a more equal contribution from all channels. A limitation in our analysis is that iEEG provides limited spatial coverage and the electrode layout is patient‐specific, making it difficult to compare patterns of band power fluctuations across subjects. To fully uncover the spatial and frequency band contributions to each dimension of each IMF, we suggest that future study should consider the spatial location of iEEG channels and perform an iterative combination of dimensionality reduction and empirical mode decomposition to find components and their contributions for each IMF. From a clinical perspective, information on the spatial coverage and location of the electrodes would further allow us to investigate the overlap of the location of these temporal fluctuations with the epileptogenic zone in focal epilepsies.

We applied empirical mode decomposition (EMD) to derive band power fluctuations on different timescales. EMD is a popular data‐driven adaptive method with applications on broad range of scientific topics, such as geology (Battista, Knapp, McGee, & Goebel, 2007), hydrology (Hu & Si, 2013) and neuroscience (Huang et al., 2013; Rojas et al., 2013) among many others. It is suitable for extracting fluctuations on different timescales without assumptions of local stationarity, linearity, or specific basis functions, and for these reasons preferable for our application. Since EMD does not require a basis function to identify different timescales of fluctuations, it also does not generate harmonics (as in Fourier or Wavelet‐type approaches) of fluctuations, making the decomposed cycles easier to interpret. However, EMD also has some limitations. Most notably, the IMFs' timescales of fluctuations may overlap, which is known as 'mode mixing' (Ur Rehman & Mandic, 2011). EMD may also struggle to distinguish two distinct fluctuations that have very similar periods, and they may be merged into one IMF. Ongoing developments (Deering & Kaiser, 2005; Li, Wang, & Zhao, 2015; Xue, Zhou, Xu, Zhu, & Li, 2015) in this area may overcome these limitations. Future study should explore how to capture nonstationary (Kaplan et al., 2005), nonlinear (Stam, 2005) and potentially hierarchical (Vidaurre, Smith, & Woolrich, 2017) time‐varying properties of the continuously recorded EEG.

Our main goal was to investigate if there is an association between variability in seizure evolutions and fluctuations in long‐term iEEG band power. Changes in seizure evolutions can be quantitatively described using seizure dissimilarity, which captures how different any pair of seizures are in a given subject in terms of their seizure network evolutions (Schroeder et al., 2020). Previous study has also shown that fluctuations in seizure evolutions were well‐explained by processes incorporating Gaussian noise, circadian, and/or slower timescales of changes in most subjects (Schroeder et al., 2020). In agreement with this study, we found that circadian or multidien fluctuations contributed strongly in most subjects in explaining seizure dissimilarity. In three subjects (ID04, ID06, ID12), the residue signal also contributed to the explanation, indicating that fluctuations on longer periods than the recording durations also played a role. Interestingly, we also found many faster (ultradian) fluctuations as explanatory variables in most subjects. These fluctuations could be contributing explanatory power through what previously was modelled as noise (Schroeder et al., 2020). However, there may also be a true biological fluctuation underpinning the explanation; faster fluctuations in the EEG have also been reported for example, in the cyclic alternating pattern (Parrino et al., 2014). With larger datasets using more seizures recorded over a longer period, future study should investigate ultradian contributions carefully and assess if noise would perform as well as the cumulative ultradian contributions.

While fluctuations in long‐term iEEG band power can explain seizure dissimilarity fairly well, this association should not be interpreted as causal evidence. The observed band power fluctuations can be understood as signatures of multiple biological processes, which could directly dictate seizure evolutions or be co‐modulated by the same upstream processes as the seizure evolutions. Our data cannot distinguish these cases. Additional fluctuations that are not captured by iEEG band power may also explain changes in seizure evolutions, and a more detailed analysis of the exact fluctuations and the differences in specific seizure features may be more informative. Interestingly, band power fluctuations did not account for all the seizure variability in most subjects. The highest adjusted *R* ^2^ was around 0.8 and the unexplained variability based on the models suggests that there are additional factors, or possibly a level of stochasticity, that impact seizure evolutions. Nevertheless, to make our findings clinically useful, for example, as a predictive model of upcoming seizure evolutions or seizure severity, neither causality nor completeness of the predictors is required. Our results indicate that a predictive model of seizure evolutions is possible with continuously recorded features such as iEEG band power, and this model should achieve good predictive performance in the majority of subjects.

To improve predictive performance, other factors could be considered in future, for example, the anti‐epileptic drug (AED) level at any given time or additional EEG features. Specifically, it is well‐known that AED changes and withdrawal can change the severity and evolutions of seizures. For example, bilateral tonic–clonic seizures are more prevalent when AED levels are reduced (Pensel et al., 2020). AEDs have further been shown to impact inter‐ and peri‐ictal brain activity (Badawy, Macdonell, Jackson, & Berkovic, 2009; Meisel et al., 2015), making it an important feature to consider. In this study, we did not incorporate information regarding drug doses, but future studies may wish to investigate how AED levels impact iEEG band power (Arzy et al., 2010), in combination with their potential explanatory power for seizure evolution changes. However, it is unlikely that AEDs are the sole driver of changing seizure characteristics, such as seizure occurrence (Karoly et al., 2021) and seizure evolution (Schroeder et al., 2020). Notably, prior studies on canine epilepsy showed that various seizure cycles (circadian, weekly and monthly) exist even in the absence of anti‐epileptic medication (Gregg et al., 2020). In future study, incorporating personalised medication records could unravel the behaviour of seizure rhythms with respect to changes in drugs and/or doses. Any multi‐way association between continuously recorded brain activity, seizure evolutions, and treatments (such as AEDs) has the potential to introduce entirely new treatment strategies. If, for example, particular interictal EEG signatures predict more severe seizures, and these signatures are also influenced by AED dose, then one can hypothesise that responsively adapting AED dose according to these interictal signatures might decrease seizure severity. If this hypothesis can be verified, then on‐demand drug‐delivery systems programmed to respond to patient‐specific interictal signatures could become the next generation of epilepsy treatments (Carney, Stanley, & Talathi, 2014; Manganaro, Loddenkemper, & Rotenberg, 2017; Ramgopal, Thome‐Souza, & Loddenkemper, 2013).

In a more general context, our study is another contribution to the wider literature of explaining ictal features from interictal EEG features or hypothesised circadian/multidien rhythms. For example, studies have established that there is often a subject‐specific relationship between fluctuations of interictal EEG features and the timing of ictal events (Baud et al., 2018; Karoly et al., 2016; Leguia et al., 2021; Maturana et al., 2020; Mitsis et al., 2020). Interestingly, we found no evidence of an association between band power fluctuations of the interictal EEG and seizure occurrence (data not shown). Seizures were not more likely to occur during particular phases of particular IMFs in most subjects in our data set. This finding is in agreement with a previous study (Mitsis et al., 2020) that reported functional network fluctuations, rather than band power fluctuations, to be more predictive of seizure timing. Future study should investigate temporal fluctuations in a range of EEG features, such as band power (Cummings et al., 2000), functional connectivity (Mitsis et al., 2020), high frequency oscillations (Gliske et al., 2018), variance and autocorrelation (Maturana et al., 2020). Apart from seizure timing, our study has shown that band power fluctuations on different timescales do explain changes in seizure evolutions. Future study should explore this avenue further to illuminate the exact processes and timescales that modulate or dictate the various aspects of a seizure.

Finally, our study contributes to the growing literature of alternative treatment approaches in epilepsy that predict and react to the temporal changes of the disease. Most prominently, predicting when seizures happen has been an active and re‐invigorated area of research for many years (Cook et al., 2013; Freestone, Karoly, & Cook, 2017; Karoly et al., 2017; Stirling, Cook, Grayden, & Karoly, 2021). Our study further contributes to being able to predict seizure dynamics and evolutions and thus also seizure severity and symptoms. Additionally, the aforementioned (slow) fluctuations in EEG features we and others investigate may also serve as biomarkers that can track treatment response, and therefore open the gateway to on‐demand treatment options (Bernard, 2021; Carney et al., 2014; Karoly, Rao, et al., 2021; LeiteGóesGitai, de Andrade, dos Santos, Attaluri, & Shetty, 2019; Potruch, Khoury, & Ilan, 2020; Ramgopal et al., 2013). The association we investigated between how seizures change and slow fluctuations in EEG features therefore serves as a vital link to make the leap between treatment outcome (improved seizure symptoms/severity) and the given intervention that can be tracked with slow fluctuations in EEG features.

In conclusion, fluctuating interictal EEG features may not only correlate with seizure timing, but also with seizure evolutions on multidien, circadian and ultradian timescales. In the future, it may be possible to use these temporal patterns of EEG fluctuations to predict seizure evolutions. Prediction of various seizure features, including seizure evolution and seizure severity is a critical unmet need for people with epilepsy. If successful, it would open up new opportunities for therapeutics and maximising quality of life.

## CONFLICT OF INTEREST

The authors declare no conflict of interest.

## ETHICS STATEMENT

The project was granted its approval by the Newcastle University Ethics Committee (Ref: 18818/2019).

## Supporting information


**APPENDIX S1**: Supporting information

## Data Availability

Patient‐specific processed data and visualizations for selected figures of the manuscript are available on Zenodo (http://dx.doi.org/10.5281/zenodo.5798022) it will be made public at point of publication.
