# Supplementary material for: Fluctuations in EEG band power at subject‐specific timescales over minutes to days explain changes in seizure evolutions
Source: Hum Brain Mapp. 2022 Feb 4;43(8):2460–77. doi: 10.1002/hbm.25796 (PMC9057101; doi:10.1002/hbm.25796)
Supplement: Supplementary file 1 — APPENDIX S1: Supporting information [file HBM-43-2460-s001.pdf]

Supporting Information

Supporting S1 Subject data information

Subject	Duration in hours	Duration in days	Number of seizures	Mean seizure duration (minutes)
**ID01**	293	12	2	10.030
**ID02**	235	10	2	1.468
**ID03**	158	7	4	1.078
**ID04**	41	2	14	0.699
**ID05**	109	5	4	0.278
**ID06**	146	6	8	0.765
**ID07**	69	3	4	1.159
**ID08**	144	6	70	0.366
**ID09**	41	2	27	0.706
**ID10**	42	2	17	1.181
**ID11**	212	9	2	1.526
**ID12**	191	8	9	2.441
**ID13**	104	4	7	1.717
**ID14**	161	7	60	0.430
**ID15**	196	8	2	1.576
**ID16**	177	7	5	3.174
**ID17**	130	5	2	1.632
**ID18**	205	9	5	3.319
**Total**	2656	111	244	
**Average**				1.864

Table Supporting S1.0.1: For each subject the following information is provided: **Duration in hours:** duration of iEEG recordings in hours. **Duration in days:** duration of iEEG recordings in days. **Number of seizures:** number of subject's seizures annotated. **Mean seizure duration:** mean seizure duration across all annotated seizures in minutes.

Supporting S2 Visualising Seizure Dissimilarity

The iEEG traces of all seizures for subject ID06 are shown in Fig. Supporting S2.0.1a for visual comparison of the different seizures and the quantified seizure evolution differences displayed as trajectories (Fig. Supporting S2.0.1b) along with the dissimilarity values (Fig. Supporting S2.0.1c) for each pair of seizures. The bottom panel of the figure is similar to Fig. 5a,b.

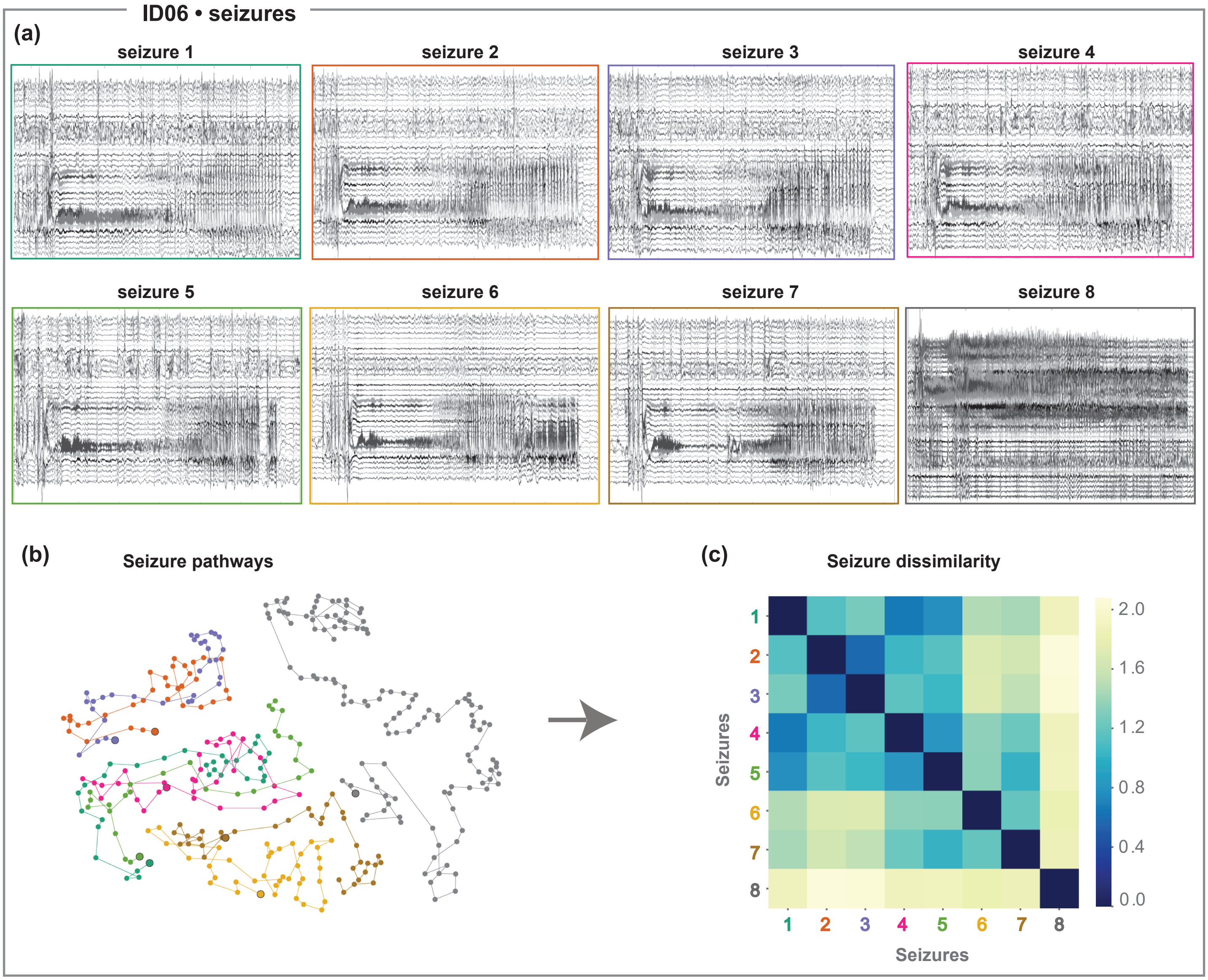

Figure Supporting S2.0.1: Seizure dissimilarity matrix for example subject ID06. (a) Seizure iEEG traces are shown in the top panel of the figure. (b) Functional network evolution of all seizures projected into 2D space using multi-dimensional scaling (MDS) for visualisation of seizure pathways (see Schroeder et al., 2020 for details). Similar seizures tend to be placed closer together in this projection. Seizures are displayed with distinct colours to distinguish seizure events. The starting points of seizures are marked with a black outline circle. (c) Seizure dissimilarity matrix, capturing the differences in seizure pathways between each pair of seizures.

Supporting S3 Association between Seizure Dissimilarity and Seizure Band Power Distance

Here, we want to demonstrate that the band power signal itself does not explain how seizure pathways change, or at least not as well as specific fluctuations of bandpower on particular timescales (as we presented in the main results). To this end, we associated each subject's pathway dissimilarity matrix with differences in the raw band power signal (termed band power distance from now on).

In order to explore if band power distance explains seizure dissimilarity, we applied a linear regression framework. We implemented two models: (i) using the seizure onset time window and (ii) using the time window just before the seizure onset (onset window - 1).

For consistency with the main part of our analysis (see Fig. 5a,b) and allow comparison with findings in Fig. 6, a pairwise band power distance was obtained as the euclidean distance from the data matrix X shown in Fig. 1b. In other words, the band power distance is the euclidean distance between two time windows in terms of their band power in all frequency bands and channels.

As can be seen in Fig. Supporting S3.0.1, the low values of adjusted R^2 across all subjects indicate the band power signal itself does not explain how seizures change over time. Instead the decomposition of the band power signal into fluctuations of particular timescales is crucial, and only some of these timescales contribute explanatory power, as we have shown in Fig. 6.

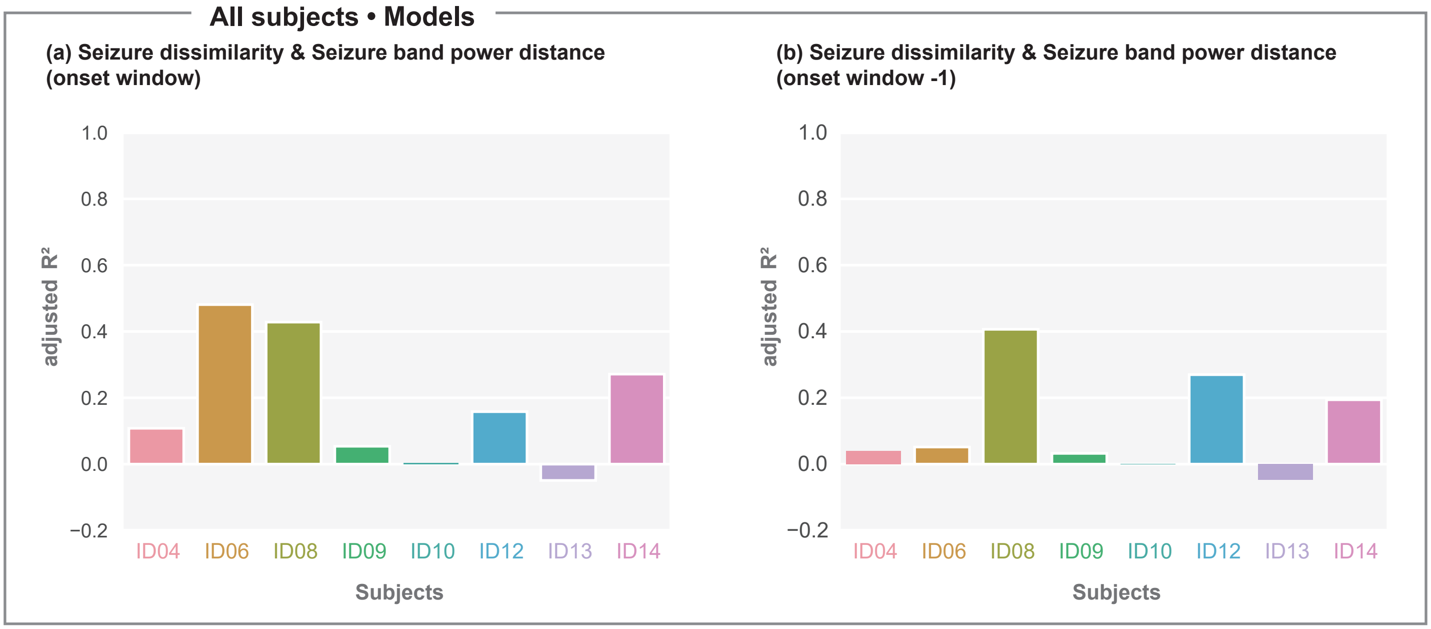

Figure Supporting S3.0.1: Relating seizure dissimilarity with seizure band power distance. (a)&(b) Summary across subjects represented with bar charts of the adjusted R^2 values obtained from the linear models using the onset window (Left plot: (a)) and the onset window -1 (Right plot: (b)).

Supporting S4 Tests for statistical significance in model R^2

Supporting S4.1 Randomly shifted onset times

We randomly shifted seizure onset times to test if the multiple linear regression model R^2 values would have occurred by chance. Adjusted R^2 values for 500 iterations, along with the actual adjusted R^2 are shown in Fig. Supporting S4.1.1.

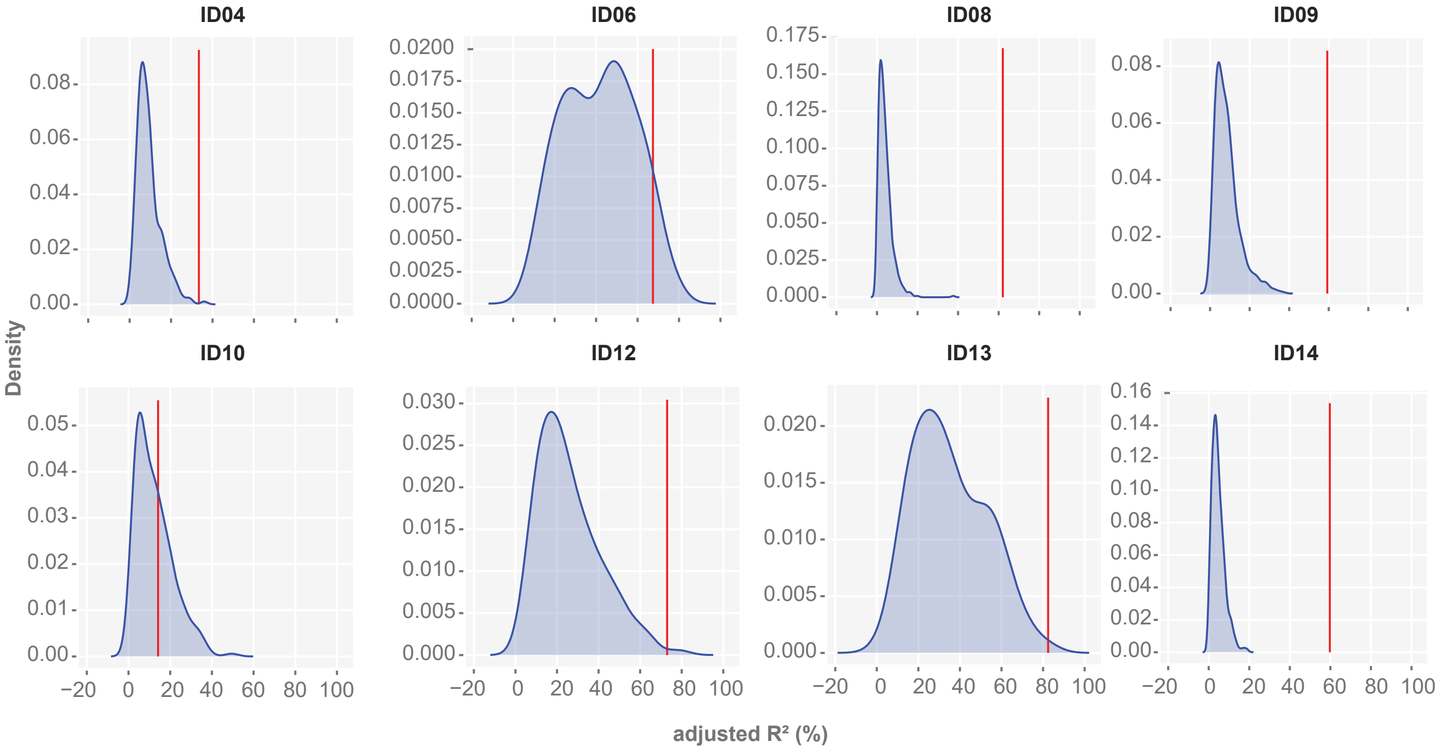

Figure Supporting S4.1.1: Distribution of the adjusted R^2 values using random seizure timings, after implementing the positive LASSO and OLS regression model for each subject. The seizure dissimilarity matrix was used as response variable, while the seizure IMF distance matrices for the random seizure times and the seizure temporal distance were included in the model as explanatory variables. The vertical red line represents the adjusted R^2 for the same analysis performed on the original seizure onset times (see Section 2.11 and Fig. 6d).

Supporting S4.2 Randomly permuted onset times

Similarly to the analysis described in the previous section, we additionally performed a permutation test. In each permutation iteration, we first randomly permuted the order of the seizures (but not their timing). Then, we obtained new IMF seizure distance matrices and performed the LASSO and linear regression, exactly as described in the Section 2.11, leaving the response variable unchanged. Finally, we calculated the adjusted R^2 for each iteration (see Fig. Supporting S4.2.1). Statistical significance was determined based on a significance level of 5%. Again, the aforementioned steps were performed for all subjects with at least six recorded seizures. Significance levels were similarly for all tested subjects as in the previous section.

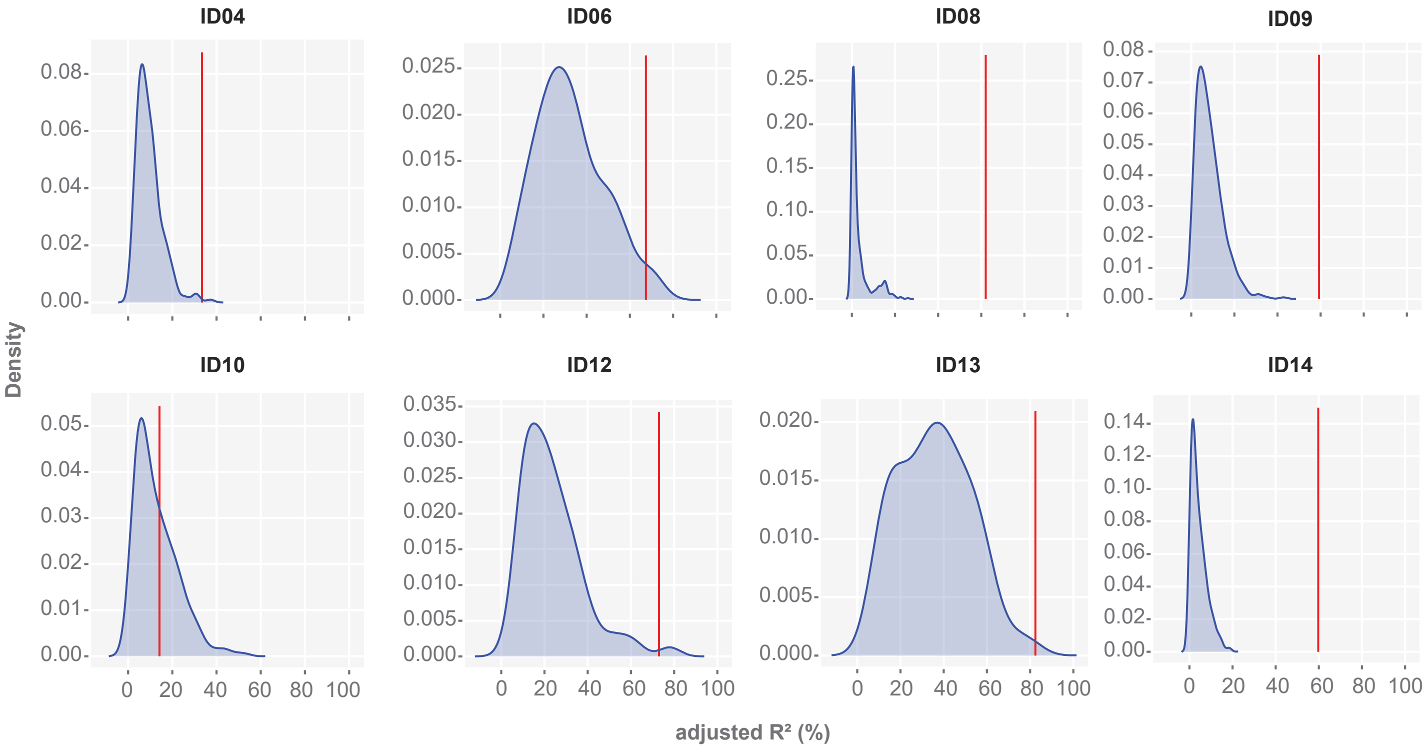

Figure Supporting S4.2.1: Distribution of the adjusted R^2 values using permuted seizure orders. The seizure dissimilarity matrix was used as response variable, while the seizure IMF distance matrices for the random seizures and the seizure temporal distance were included in the model as explanatory variables. The vertical red line represents the adjusted R^2 for the same analysis performed on the original seizure order (see Section 2.11 and Fig. 6d).

Supporting S5 Gini index for frequency bands θ, α, β and γ

As described in Section 2.8, we obtained the Gini index for the frequency bands: θ, α, β and γ (see Fig. Supporting S5.0.1) in the same way as for δ band (Fig. 4).

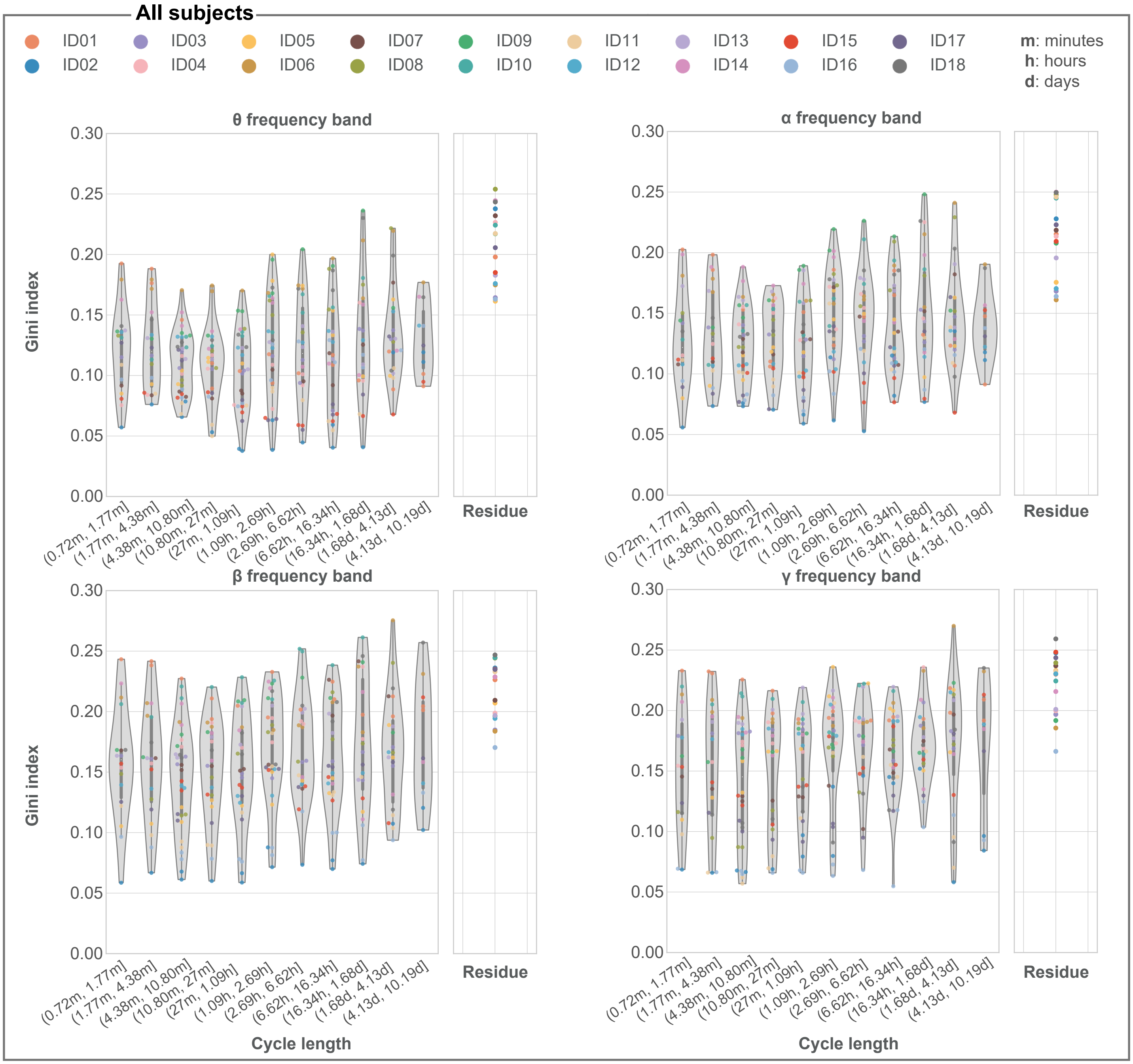

Figure Supporting S5.0.1: Supporting Gini Index results for the θ, α, β and γ frequency bands across all subjects. Equivalent figure to Fig. 4

Supporting S6 Choice of tuning parameter λ for LASSO

Figures Supporting S6.0.1 & Supporting S6.0.2 are complementary plots supporting the intermediate steps of the analysis described in Section 2.11. We implemented a 10-fold cross validation for choosing the best λ parameter in LASSO. We chose a λ that minimize the Cross-Validation Mean Square Error (CV-MSE) for the validated data set. The CV-MSE error for the training data set is also presented in Fig. Supporting S6.0.1 for reference for one example subject, ID06.

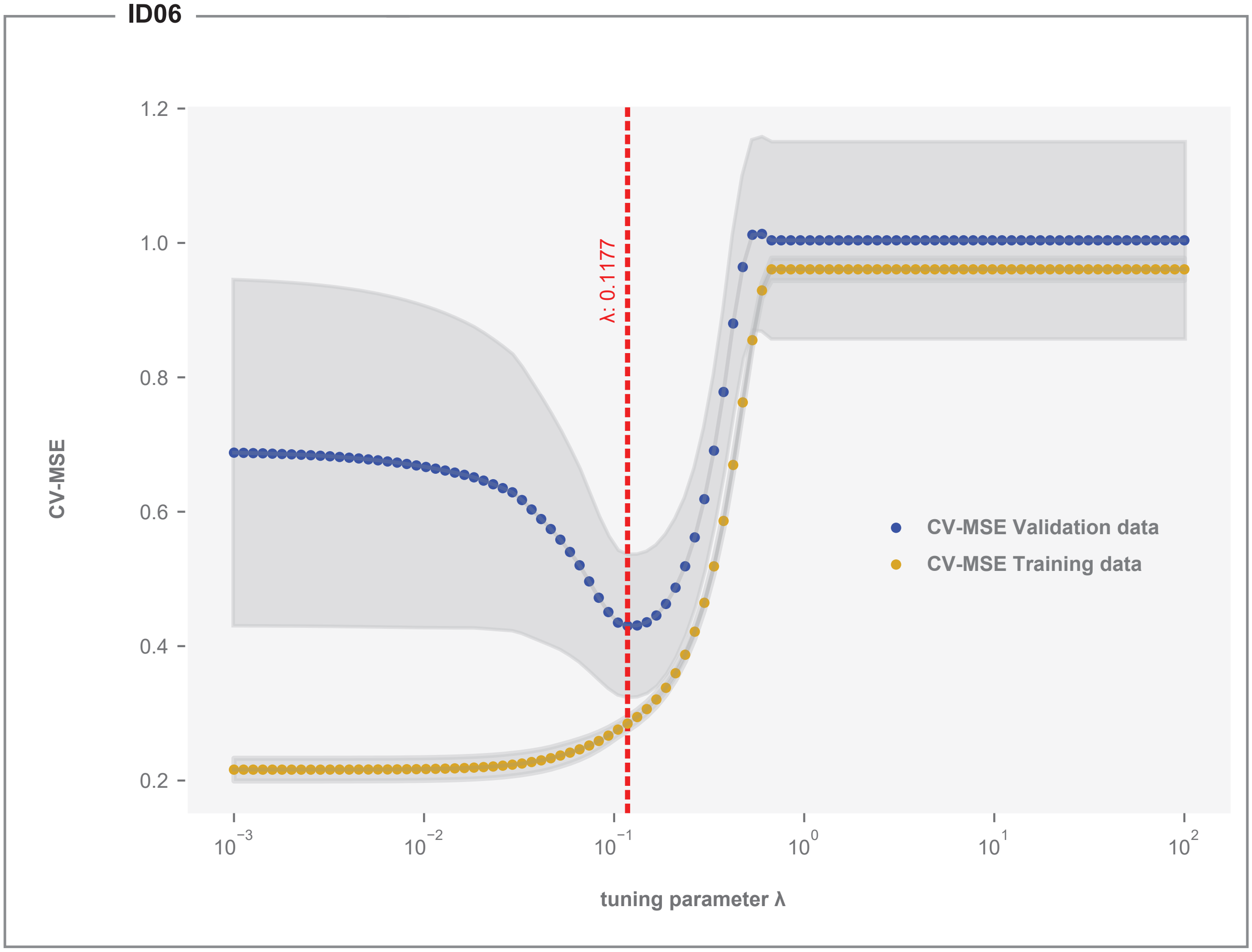

Figure Supporting S6.0.1: Cross-validation MSEs for the application of positive LASSO regression for example subject ID06. For each value of the tuning parameter λ, the CV-MSEs across the 10 folds are displayed in blue along with error bars which cover the mean plus or minus one standard error. Training MSE is displayed in yellow. The red vertical line represents the selected λ value that corresponds to the minimum Cross-Validation MSE for the validated data set.

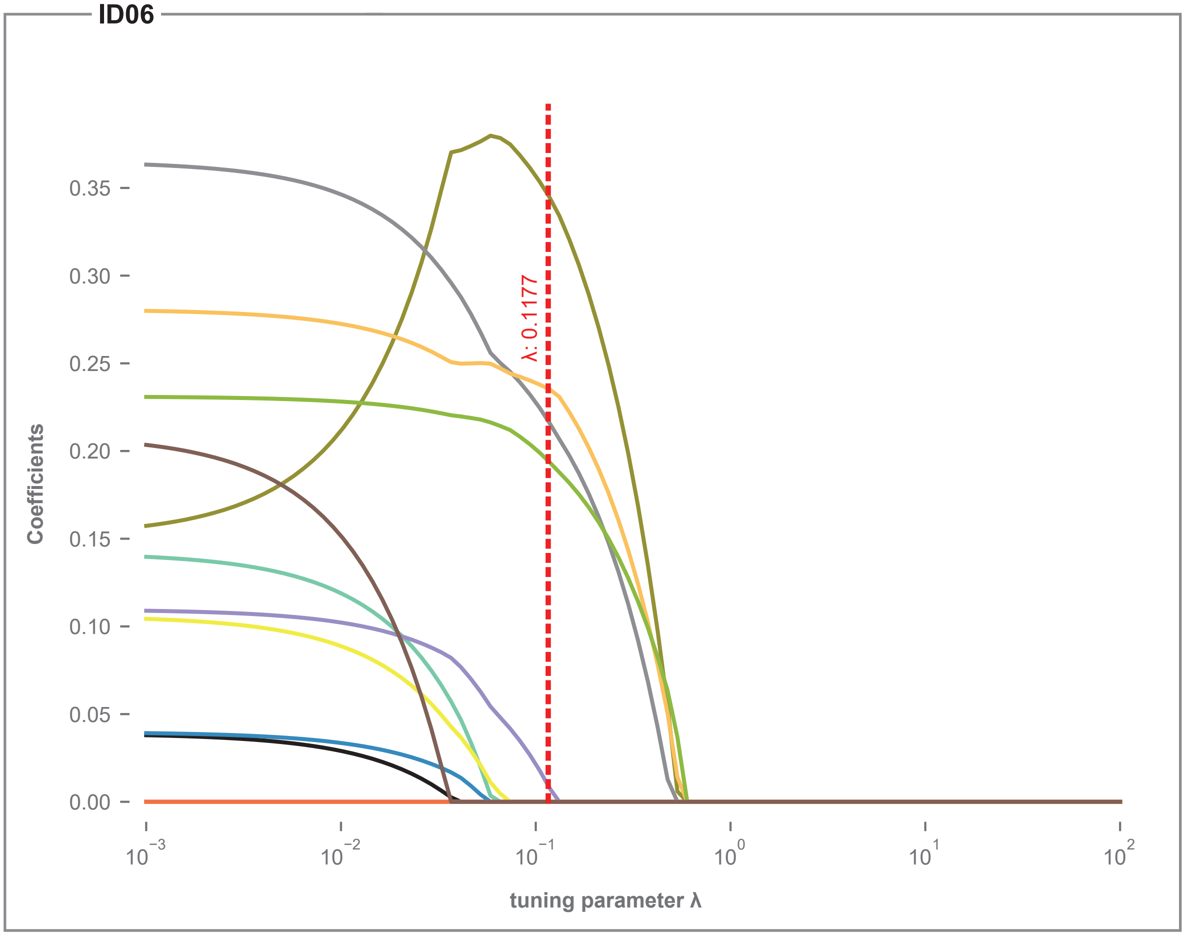

Figure Supporting S6.0.2: The effect of the tuning parameter λ on positive LASSO regression coefficients for subject ID06. Each line represents the regression coefficient estimate for each explanatory variable. The red vertical line corresponds to the λ parameter selected based on 10-fold cross validation approach.

Supporting S7 Determining which IMF fluctuations overlap with noise

In order to evaluate if each of the IMFs is a good representation of fluctuations present in the data, we implemented an empirical analysis based on permutation resampling of the original time series (H expression coefficients). We used this nullmodel to produce distributions of IMF fluctuation frequencies that would be expected from noise. In our nullmodel, we assumed a permuted time series (permuting time points in H) to represent noise. I.e. we preserve the distribution of the values in the time series, but the temporal fluctuations are destroyed through the permutation.

We randomly shuffled the columns (timing) of the H matrix over 50 iterations and performed the MEMD analysis for each iteration. Then, for each IMF, in each iteration, we estimated the 2D distribution of the instantaneous frequency, and instantaneous amplitude (across all time points). We formed the average distribution across all iterations. We repeated the same analysis for the original (non-shuffled) data, for each IMF. Therefore we can calculate overlap in the distributions (between original and shuffled data).

We used a 2D grid of (frequency, amplitude) with 800 frequency bin between 10^{-3} to 10^{4} in logarithmic scale, and 400 amplitude bins between 10^{-4} to 10^{0} in logarithmic scale (logarithmic scales of base 10 were used). In each (frequency, amplitude) bin, where the original signal overlapped with the permuted signal, the corresponding data points were labelled as overlapping with noise. These points can subsequently be removed from the calculation of the marginal Hilbert-Huang spectra for the original signal. These newly obtained marginal Hilbert-Huang spectra, excluding data points overlapping with noise, are shown in red in (Fig. Supporting S7.0.1 & Supporting S7.0.2).

In Fig. Supporting S7.0.1 & Supporting S7.0.2, we can clearly see that many frequencies of fluctuations in IMF1-3 are overlapping with noise in most subjects. The slower IMFs do not appear to be affected (as noise-IMF tend to decrease in amplitude for slower IMFs). While we could discard faster IMFs as noise due to the overlap, it is worth noting that these faster IMFs could carry some true fluctuation that is simply on the same timescale and of the same amplitude as the noise. This would be impossible to distinguish here, and therefore we present all results on all IMFs in the main text and will present supporting results with the faster IMFs removed in Supporting S8.2.

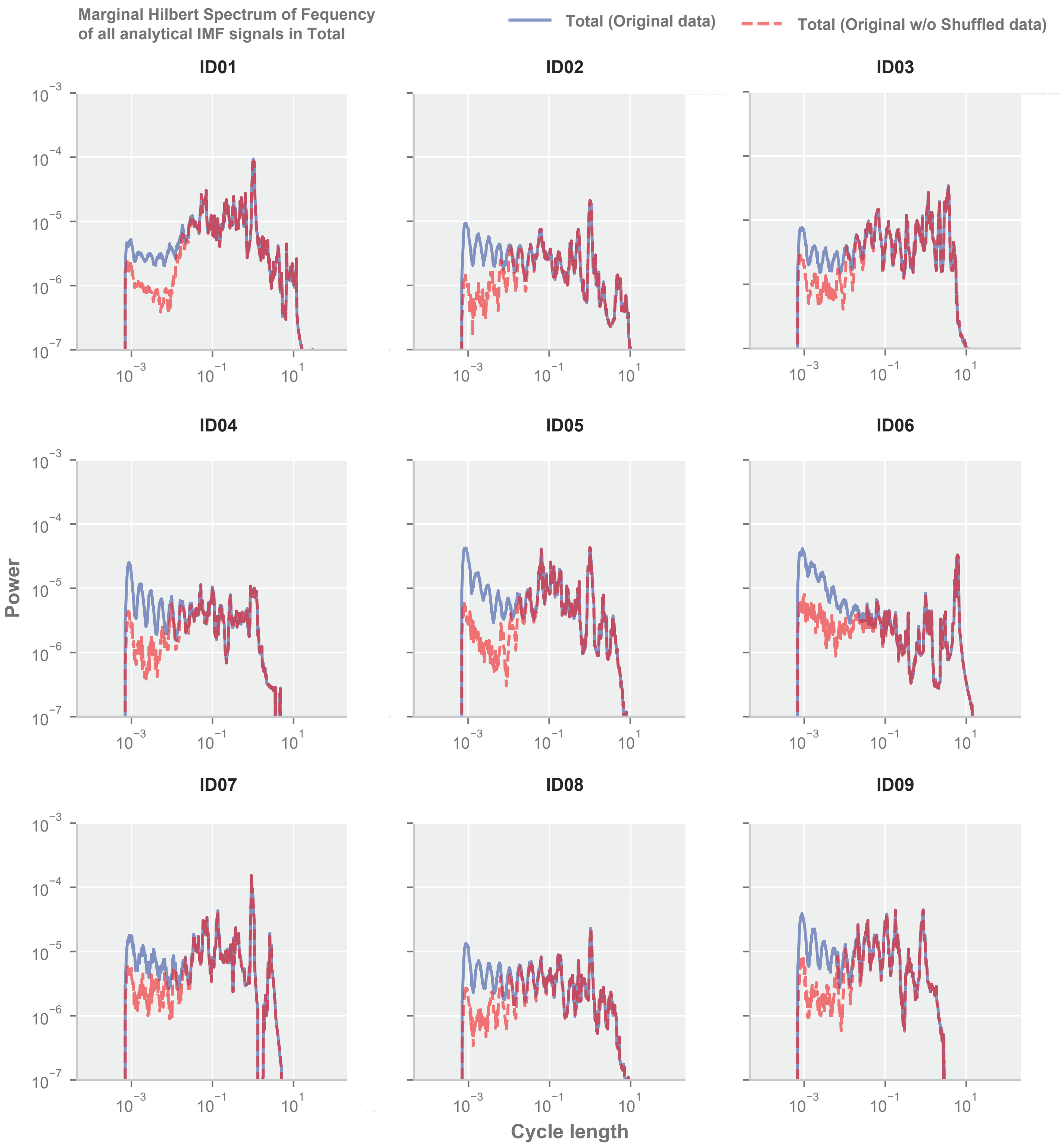

Figure Supporting S7.0.1: Marginal Hilbert spectrum of frequency for all analytical IMF signals across all dimensions for both the original data (blue line) and after excluding frequency-amplitude data points overlapping with noise (red line). Each panel corresponds to one subject.

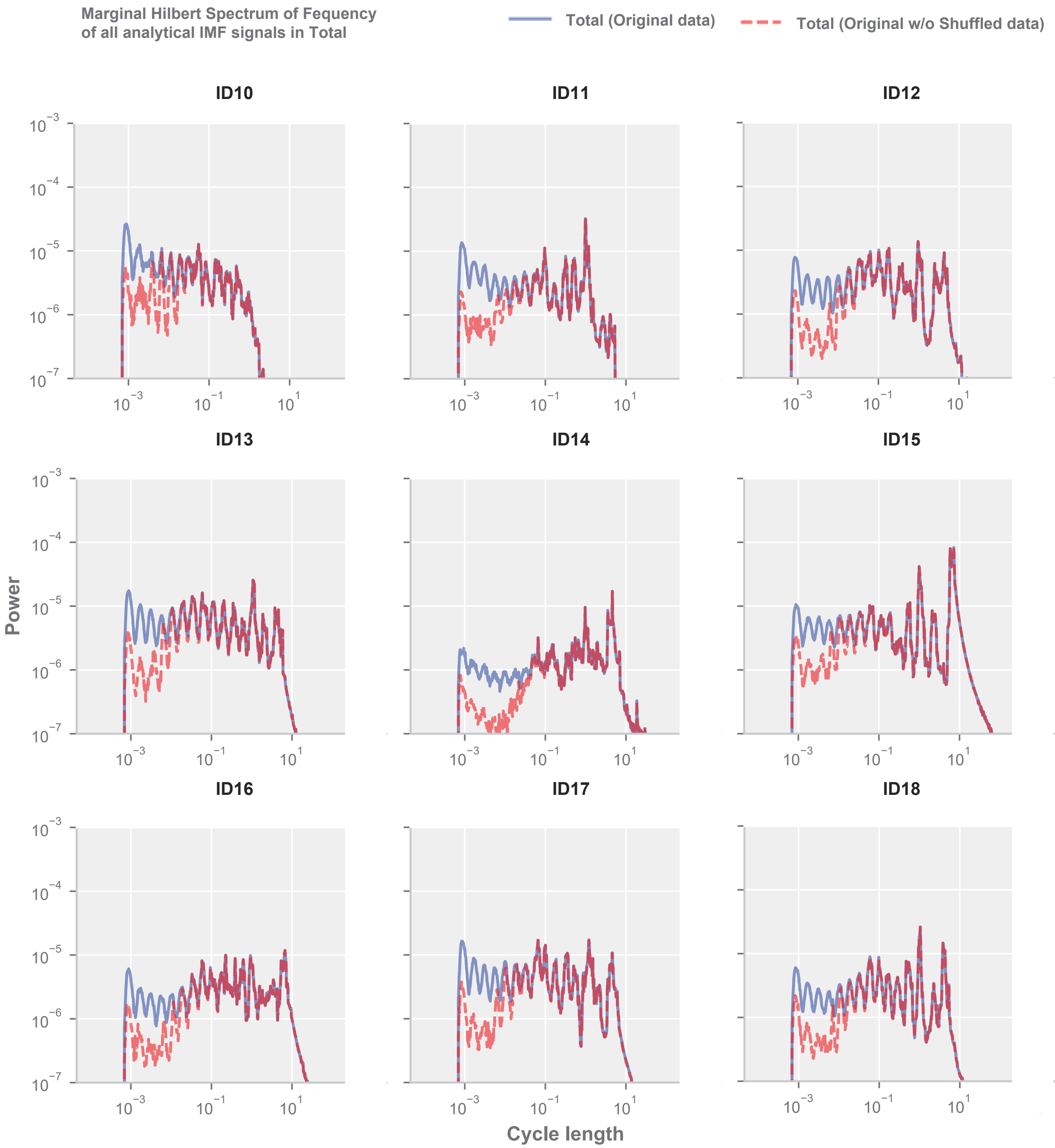

Figure Supporting S7.0.2: Continued: Marginal Hilbert spectrum of frequency for all analytical IMF signals across all dimensions for both the original data (blue line) and after excluding frequency-amplitude data points overlapping with noise (red line). Each panel corresponds to one subject.

Supporting S8 Alternative models for explaining the diversity in within-subject seizure evolutions

We tested additional models to see how well they explain seizure variability, using the same framework as is described in Section 2.11.

Supporting S8.1 Association between seizure dissimilarity & IMF seizure distance based on the time window before the seizure

To further validate our model in terms of the time window chosen for obtaining the seizure IMF distances, we performed an additional analysis using one time window before the window containing the seizure onset (termed onset window-1). The reasoning is that the IMF distances obtained in this manner cannot be containing any seizure-related changes in band power. As can be seen in Fig. Supporting S8.2.1a and in the first two columns of Table Supporting S8.2.1, the adjusted R^2, as well as the coefficient estimates and the IMF components remaining in the model for each subject are in agreement with the model shown in Fig. 6d. Thus, both models perform similarly indicating that the IMF distance results are robust towards changing a single window.

Supporting S8.2 Association between seizure dissimilarity & IMF seizure distance excluding noise

We further performed the regression analysis in Section 2.11 excluding the first three IMFs, which could represent noise (Supporting S7). As can be seen in Fig. Supporting S8.2.1, the adjusted R^2 values for the majority of subjects were comparable for the two models (albeit generally slightly lower). Only for subjects ID09, the adjusted R^2 was dramatically lower for the model without the first three IMFs (see Table Supporting S8.2.1 and Fig. Supporting S8.2.1b). Note also our Discussion on the role of the faster IMFs.

We also observed that the IMFs and corresponding coefficients were substantially different in ID04, ID09, and ID10 between the two models. This is not surprising given that both ID04 and ID10 had a low adjusted R^2 in the first place, and ID09 had a low adjusted R^2 in the model without the first IMFs. In summary, we conclude that the first three IMFs do not contribute substantially to explaining seizure dissimilarities in most subjects. However, in some subjects, faster IMFs may play a strong role in explaining seizure dissimilarities.

subjects	Seizure dissimilarity & IMF Seizure distance Adjusted R^2		
	onset window	onset window -1	onset window w/o noise
**ID04**	0.3343	0.1545	0.0347
**ID06**	0.6742	0.5755	0.6787
**ID08**	0.6207	0.6279	0.6201
**ID09**	0.5927	0.5601	0.1865
**ID10**	0.1419	0.1949	0.0608
**ID12**	0.7299	0.6489	0.6529
**ID13**	0.8243	0.8060	0.7197
**ID14**	0.5973	0.5834	0.5306

Table Supporting S8.2.1: Adjusted R^2 values for the models described in Sections 2.11, Supporting S8.1 & Supporting S8.2 for each subject with at least six recorded seizures.

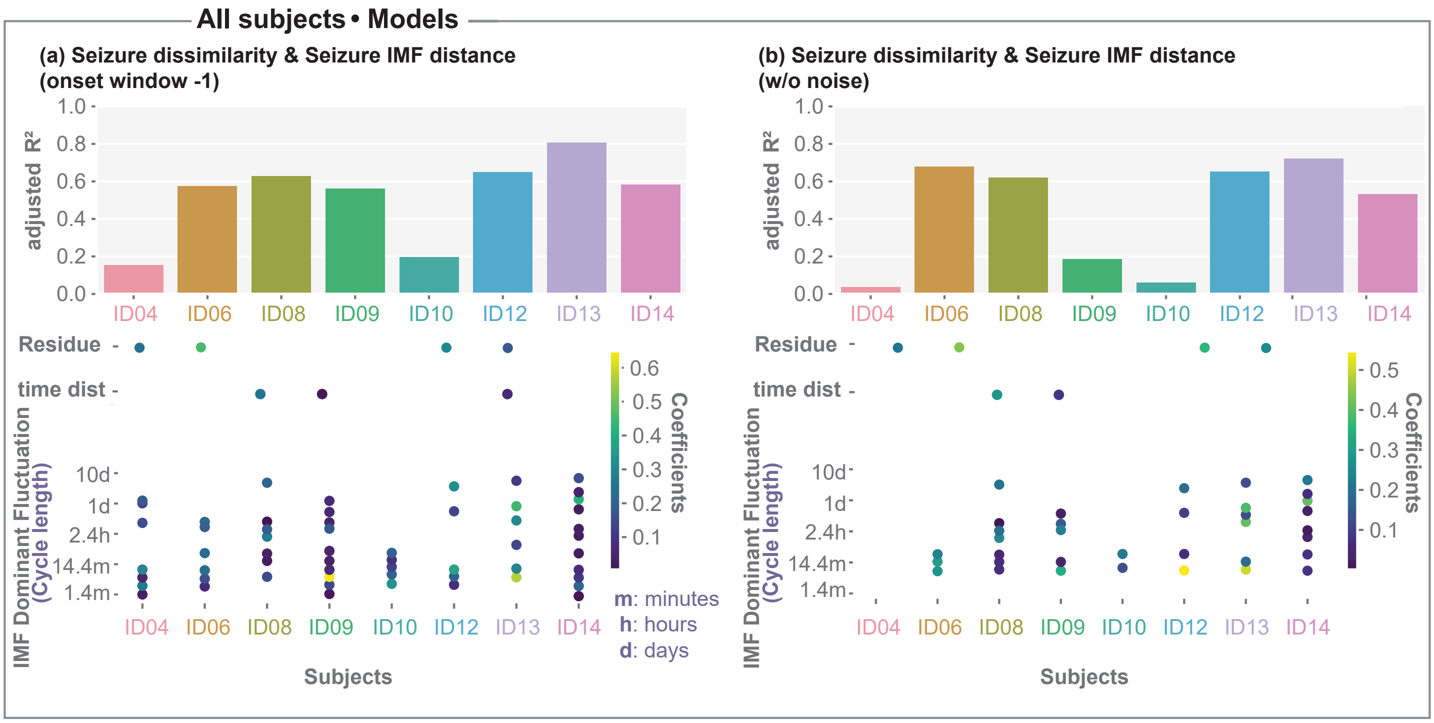

Figure Supporting S8.2.1: (a)&(b) Summary across subjects based on OLS models with explanatory variables obtained by the constrained LASSO using similar representation as in Fig.6d for the models described in Section Supporting S8.1 (Left plot: (a)) and Section Supporting S8.2 (Right plot: (b)). Top: Bar chart of the adjusted R^2. Bottom: Dot plot indicating the OLS coefficient estimates for the residue or time distance (when this variable remained in the model) together with OLS coefficient estimates at the corresponding value of IMF peak frequency for each subject. For visualisation, we converted the peak frequency to cycle length.
